# Conformation of the Solute-Binding Protein AdcAII Influences Zinc Uptake in *Streptococcus pneumoniae*

**DOI:** 10.3389/fcimb.2021.729981

**Published:** 2021-08-19

**Authors:** Marina L. Župan, Zhenyao Luo, Katherine Ganio, Victoria G. Pederick, Stephanie L. Neville, Evelyne Deplazes, Boštjan Kobe, Christopher A. McDevitt

**Affiliations:** ^1^Department of Microbiology and Immunology, The Peter Doherty Institute for Infection and Immunity, University of Melbourne, Melbourne, VIC, Australia; ^2^School of Chemistry and Molecular Biosciences, The University of Queensland, Brisbane, QLD, Australia; ^3^Australian Infectious Diseases Research Centre, The University of Queensland, Brisbane, QLD, Australia; ^4^Institute for Molecular Bioscience, The University of Queensland, Brisbane, QLD, Australia; ^5^Department of Molecular and Biomedical Science, School of Biological Sciences, The University of Adelaide, Adelaide, SA, Australia; ^6^School of Life Sciences, University of Technology Sydney, Ultimo, NSW, Australia; ^7^School of Pharmacy and Biomedical Sciences, Curtin Health Innovation Research Institute, Curtin University, Bentley, WA, Australia

**Keywords:** *Streptococcus pneumoniae*, zinc, solute-binding protein, ABC transporter, host-pathogen

## Abstract

*Streptococcus pneumoniae* scavenges essential zinc ions from the host during colonization and infection. This is achieved by the ATP-binding cassette transporter, AdcCB, and two solute-binding proteins (SBPs), AdcA and AdcAII. It has been established that AdcAII serves a greater role during initial infection, but the molecular details of how the protein selectively acquires Zn(II) remain poorly understood. This can be attributed to the refractory nature of metal-free AdcAII to high-resolution structural determination techniques. Here, we overcome this issue by separately mutating the Zn(II)-coordinating residues and performing a combination of structural and biochemical analyses on the variant proteins. Structural analyses of Zn(II)-bound AdcAII variants revealed that specific regions within the protein underwent conformational changes *via* direct coupling to each of the metal-binding residues. Quantitative *in vitro* metal-binding assays combined with affinity determination and phenotypic growth assays revealed that each of the four Zn(II)-coordinating residues contributes to metal binding by AdcAII. Intriguingly, the phenotypic growth impact of the mutant *adcAII* alleles was, in general, independent of affinity, suggesting that the Zn(II)-bound conformation of the SBP is crucial for efficacious metal uptake. Collectively, these data highlight the intimate coupling of ligand affinity with protein conformational change in ligand-receptor proteins and provide a putative mechanism for AdcAII. These findings provide further mechanistic insight into the structural and functional diversity of SBPs that is broadly applicable to other prokaryotes.

## Introduction

Zinc is an essential micronutrient in all domains of life. Zinc, which occurs as the divalent cation Zn(II), fulfils both structural and functional roles within proteins, and is estimated to be required by up to 6% of the bacterial proteome (Andreini et al., 2006). Zinc serves in numerous essential cellular processes, such as DNA transcription and central carbon metabolism. Accordingly, Zn(II) acquisition is crucial for bacterial growth and virulence (Boylan et al., 2003; Hantke, 2005). The mammalian host exploits the essentiality of Zn(II) to control infection, by manipulating Zn(II) abundance and bioavailability (Hennigar and McClung, 2016). Nutritional immunity is the process wherein nutrients essential to infection by pathogens are withdrawn or have their bioavailability restricted. For Zn(II), this can be achieved by proteins such as human serum albumin (Lu et al., 2008; Cerasi et al., 2013) or the S100 family protein calprotectin, which is released from neutrophils (Kehl-Fie and Skaar, 2010). To overcome host-imposed Zn(II) restriction, pathogenic bacteria have evolved high-affinity acquisition systems to compete for metal ions at the host-pathogen interface (Ammendola et al., 2007; Sabri et al., 2009; Bayle et al., 2011; Pederick et al., 2015). These include the ubiquitous Type II ATP-binding cassette (ABC) family importers (Lewis et al., 2012), zinc-iron permease (ZIP) transporters (Grass et al., 2005; Karlinsey et al., 2010), P-type ATPases (Lewinson et al., 2009; Chien et al., 2013), and the recently identified zincophore scavenging systems (Lhospice et al., 2017; Morey and Kehl-Fie, 2020). Bacterial ABC permeases employ extra-cytoplasmic solute-binding proteins (SBPs) to obtain cargo such as Zn(II) ions from the periplasm (Gram-negative bacteria) or the extracellular environment (Gram-positive bacteria) (Lewis et al., 2012).

*Streptococcus pneumoniae* (the pneumococcus) is a leading bacterial cause of pneumonia and meningitis and is estimated to cause more than one million deaths annually (Weiser et al., 2018). As an obligate human pathogen, the pneumococcus is dependent on the host for nutrients (Henriques-Normark and Normark, 2010). The micronutrient Zn(II) is crucial for the pneumococcus to colonize and mediate disease in host tissues. Deletion of the Zn(II)-uptake machinery severely attenuates pneumococcal virulence (Plumptre et al., 2014). In *S. pneumoniae*, Zn(II) is primarily acquired *via* the ABC transporter AdcCB, which is comprised of a transmembrane domain (AdcB) and a nucleotide binding/hydrolyzing domain (AdcC), and two cluster A-I SBPs, AdcA and AdcAII (Dintilhac et al., 1997; Loisel et al., 2008). Both AdcA and AdcAII deliver Zn(II) to AdcCB, with either SBP sufficient for *in vitro* Zn(II) uptake (Bayle et al., 2011). However, murine studies show that both SBPs are required for full virulence *in vivo* with AdcAII contributing to a greater extent at the early stages of infection and invasive disease (Plumptre et al., 2014; Brown et al., 2016). Expression of the pneumococcal Zn(II) uptake machinery is principally controlled by the Zn(II)-dependent negative regulator AdcR, albeit to different extents (Plumptre et al., 2014). Notably, AdcAII is more highly upregulated than AdcA during growth in Zn(II)-restricted conditions (Reyes-Caballero et al., 2010; Plumptre et al., 2014). This is attributable to the distinct genetic location of *adcAII* and the presence of two AdcR binding sites upstream of the gene (Plumptre et al., 2014). AdcAII is also co-expressed with the cell wall-localized Zn(II)-binding polyhistidine triad (Pht) proteins (Loisel et al., 2008; Plumptre et al., 2014). Studies show that AdcAII functions cooperatively with the Pht proteins to recruit Zn(II), but the mechanistic basis for how this occurs remains to be defined (Loisel et al., 2011; Bersch et al., 2013). Collectively, these observations have led to the proposal that AdcAII enhances Zn(II) uptake in metal-deficient niches, thereby aiding pneumococcal colonization and dissemination (Plumptre et al., 2014).

AdcAII belongs to the cluster A-I subgroup of SBPs, consisting of a conserved structural fold of two (β/α)_4_ domains connected by a rigid α-helix, with the metal-binding site located at the interface between the N- and C-terminal lobes (Loisel et al., 2008; Yukl, 2017). Although both AdcA and AdcAII share this conserved fold, AdcA contains additional structural elements associated with Zn(II) recruitment (Plumptre et al., 2014). These are an extended C-terminal domain, which has homology to the Gram-negative Zn(II) chaperone ZinT, and a histidine-rich loop, which is common in Zn(II)-specific SBPs (Plumptre et al., 2014; Luo et al., 2021). AdcAII lacks these regions and although it does have a flexible loop of residues near the metal binding site, this loop is devoid of histidine residues (Wei et al., 2007; Loisel et al., 2008; Petrarca et al., 2010). Previous crystallographic studies of Zn(II)-specific SBPs from both Gram-positive and Gram-negative bacteria have shown only minor structural differences between the metal-free and Zn(II)-bound states (Chandra et al., 2007; Yatsunyk et al., 2008; Neupane et al., 2017; Luo et al., 2021). However, whether this fully represents the mechanistic diversity employed by Zn(II)-recruiting SBPs remains to be ascertained.

In this study, we investigated the Zn(II)-binding mechanism of AdcAII by mutating each of the four Zn(II)-coordinating residues in the SBP. The AdcAII mutant variants were then analyzed using a combination of biochemical, structural and molecular microbiology approaches. X-ray crystallographic analyses and molecular dynamics (MD) simulations showed that AdcAII samples a larger conformational landscape than other Zn(II)-specific SBPs reported to date. The binding site mutations impacted protein conformation and interaction with Zn(II) to differing extents. Surprisingly, phenotypic growth analyses of the AdcAII variants showed that perturbation of the Zn(II)-bound SBP conformation had a greater impact on *S. pneumoniae* growth in Zn(II)-limited media than relative affinity for Zn(II). Taken together, these data demonstrate the importance of SBP-metal complex conformation for bacterial Zn(II) uptake. Collectively, this study advances our mechanistic understanding of ligand recruitment by SBPs and uptake *via* the associated ABC transporter. This knowledge provides a foundation for future structure-based design of novel inhibitors targeting metal-ion uptake pathways.

## Methods and Materials

### AdcAII Conservation Analysis

A global database of 20,020 publicly available *S. pneumoniae* genome sequences derived from the Global Pneumococcal Sequencing project (www.pneumogen.net) (Gladstone et al., 2019) was assembled by the Wellcome Sanger Institute Pathogen Informatics pipeline (Page et al., 2016). The *Streptococcus pneumoniae* serotype 2 D39 genome (NCBI identifier NC_008533) served as the reference genome to determine the presence, amino acid sequence and alignment of spd_0888 (AdcAII) across the 20,020 clinical isolates using the screen_assembly script (Davies et al., 2019) and BLASTN v2.9.0 with parameters of 80% coverage and 80% identity. For PspC and PspA, parameters of 50% coverage and 50% identity were used. Amino acid variation was determined using MUSCLE alignment in Geneious Prime (Kearse et al., 2012). Sequence conservation was determined by percentage of variant amino acids compared to consensus. Sequence conservation percentage was plotted against residue number in GraphPad Prism (Version 8). The alignment of non-redundant sequences was used to map residue conservation percentage onto Zn(II)-bound AdcAII (PDB ID: 3CX3) in UCSF Chimera *via* “render by conservation”.

### Bacterial Strains, Culturing and Growth Experiments

The mutant strain D39 Δ*adcA* was generated previously (Plumptre et al., 2014). Mutant strains in which wild-type *adcAII* was replaced with mutant variants were constructed using the Janus cassette (Sung et al., 2001). Briefly, the upstream and downstream flanking regions of *adcAII* were amplified using primers (**Supplementary Table 6**) with complementarity to the Janus cassette and were joined to the Janus cassette by overlap extension PCR. These linear fragments were used to replace *adcAII* in the chromosome of D39 Δ*adcA* mutant strains by homologous recombination. Successful transformants were identified *via* negative selection by virtue of growth on kanamycin and sensitivity to streptomycin. This strain, denoted D39 Δ*adcA* Δ*adcAII*, was subsequently used to generate *adcAII* binding site mutants of *S. pneumoniae*. Exchange of the Janus cassette with mutant variants of *adcAII* was achieved by homologous recombination of mutant *adcAII* inserts previously combined with the upstream and downstream flanking regions of genomic *adcAII* by overlap extension PCR. *S. pneumoniae* wild-type and mutant strains (**Supplementary Table 5**) were routinely grown at 37°C with 5% CO_2_ on blood agar (BA) [39 g/L Columbia base agar (Oxoid), 5% defibrinated horse blood] or THY [Todd-Hewitt broth (Oxoid), 0.5% w/v Bacto yeast extract]. Growth media were supplemented with antibiotics where appropriate: chloramphenicol (6 µg.mL^-1^), streptomycin (150 µg.mL^-1^), or kanamycin (50 µg.mL^-1^). *E. coli* strains were routinely grown at 37°C in Luria Bertani Lennox broth (LB) or on LB 1.5% agar plates supplemented with kanamycin (50 µg.mL^-1^) to maintain plasmids. For growth kinetic assays, *S. pneumoniae* strains were grown in a chemically defined medium (CDM; Dulbecco’s modified Eagle medium base, 1 × BME vitamins, 74 µM adenine, 89.2 µM uracil, 65.7 µM xanthine, 66.2 µM guanine, 1123.5 µM D,L-alanine, 757 µM L-asparagine, 1127 µM L-aspartic acid, 684.5 µM L-glutamine, 1019.5 µM L-glutamic acid, 868.6 µM L-proline, 734.9 µM L-tryptophan, 4125.4 µM L-cysteine, 12 µM lipoic acid, 1 µM pyruvate; pH 7.4). Metal content of CDM was determined by ICP-MS, as described previously (Neville et al., 2020). CDM routinely contained <1 µM Zn(II) and <0.1 µM Mn(II). *S. pneumoniae* strains were grown to mid-log (OD_600_ = 0.3) in CDM supplemented with 1 µM MnSO_4_ and 10 µM ZnSO_4_. Strains were then sub-cultured into 96-well microtiter plates to a final OD_600_ of 0.05 in 200 µL of CDM supplemented with varying concentrations of ZnSO_4_. Microtiter plates were sealed with gas permeable seals (Sigma Aldrich) and incubated at 37°C, 5% CO_2_ in a CLARIOstar spectrophotometer (BMG Labtech), with measurements (OD_600_) recorded every 30 min for 16 h.

### Whole Cell Metal Ion Accumulation Analyses

Whole cell metal ion accumulation was determined by ICP-MS essentially as previously described (Plumptre et al., 2014). Briefly, *S. pneumoniae* strains were grown to mid-log phase (OD_600_ = 0.3) in C+Y (per litre; 5 g yeast extract, 5 g casein hydrolysate, 6 mg L-tryptophan, 35 mg L-cysteine, 2 g sodium acetate, 8.5 g K_2_HPO_4_, 0.5 g MgC1_2_.6H_2_0, 2.5 mg CaC1_2_, 0.2 µg biotin, 0.2 mg nicotinic acid, 0.2 mg pyridoxine-HC1, 0.2 mg thiamine-HC1, 0.1 mg riboflavin, 0.6 mg calcium pantothenate, 2 g glucose, and 12 mL 4% bovine serum albumin) supplemented with 1 µM MnSO_4_ and 10 µM ZnSO_4_. Following three washes with 5 mM EDTA PBS and three washes with PBS, cells were harvested by centrifugation at 7,000 × *g* for 10 min and desiccated at 96°C for 18 h. Pellets were digested by treatment with 200 µL of 65% HNO_3_ at 96°C for 10 min, then diluted in MilliQ for metal-content analyses on an Agilent 8900 ICP-QQQ (Bio21, Melbourne).

### Expression and Purification of Recombinant AdcAII and Mutant Variants

Recombinant AdcAII (residues 35 – 311) was cloned from *S. pneumoniae* D39 genomic DNA (gene SPD_0888) into pCAMcLIC01 *via* ligation independent cloning as previously described (McDevitt et al., 2011; Eijkelkamp et al., 2014). Mutant variants were generated by site-directed mutagenesis (QuikChange Lightning Kit, Agilent Technologies) using primers listed in **Supplementary Table 6**. Wild-type and mutant AdcAII variants were expressed in *E. coli* LEMO21(DE3) from their respective expression constructs (**Supplementary Table 7**). Expression cultures were grown in Overnight Express Instant TB medium (Merck) supplemented with 1% glycerol and 100 µg.mL^-1^ kanamycin for 18 h at 27°C. *E. coli* pellets were resuspended in 50 mM MOPS (pH 7.2), 200 mM NaCl, 20% glycerol, 15 mM imidazole buffer. Cells were homogenized with two Complete EDTA-free protease cocktail inhibitor tablets per 0.5 L of cells. Cells were mechanically disrupted using a Constant Systems Cell Disruptor at 30 kPSI. Cellular debris and membranes were removed *via* ultracentrifugation at 120,000 × *g* at 4°C for 1 h. Recombinant proteins were then purified *via* immobilized metal ion affinity chromatography. Soluble histidine-tagged protein was purified *via* three 5 mL HisTrap HP columns (GE Healthcare) connected in tandem. The dodecahistidine tag was removed from affinity-purified protein by enzymatic digestion by the human rhinovirus 3C protease and the protein was purified further on a HisTrap HP column. Metal-free protein was prepared by dialyzing the dodecahistidine tag-cleaved protein in a 20 kDa molecular weight-cutoff (MWCO) membrane (Slide-A-Lyzer, ThermoFisher Scientific) against 4 L of sodium acetate (pH 5) buffer with 50 mM EDTA at 25°C for 24 h. The sample was then dialyzed against 4 L of 50 mM MOPS (pH 7.2), 200 mM NaCl buffer at 4°C for 24 h and centrifuged at 18,000 × *g* for 15 min to remove insoluble material. Protein samples were analyzed for metal content by heating 5 µM protein at 96°C for 15 min in 3.5% HNO_3_ with metal-ion content measured by ICP-MS. Protein samples were ultracentrifuged at 204,428 × *g* for 20 min to remove aggregating protein prior to biochemical analyses.

### Metal-Loading Analyses

Metal-free AdcAII (10 µM; <10% metal content) was incubated with a molar excess of each metal – 10-fold for MnSO_4_, CoCl_2_, NiCl_2_ and ZnSO_4_, and a 5-fold excess of CuSO_4_. Incubation of AdcAII with 10-fold for Cu(II) initially showed more than 2 Cu(II) atoms per molecule, despite AdcAII possessing only a single binding site. Accordingly, the molar excess of Cu(II) used in loading assays was reduced to minimize non-specific binding. Metal-loading assays were performed in 50 mM MOPS (pH 7.2) and 100 mM NaCl in a total volume of 2 mL, with samples incubated at 25°C for 30 min with agitation. Unbound metals were removed by desalting on a PD10 column (GE Healthcare). The metal:protein molar ratio was determined by ICP-MS using established methods (McDevitt et al., 2011).

### Differential Scanning Fluorimetry

Metal-free AdcAII (10 µM; <10% metal content) was incubated with a 10-fold molar excess of MnSO_4_, FeSO_4_, CoCl_2_, NiCl_2_, CuSO_4_ or ZnSO_4_ at 25°C in the presence of 5 × SYPRO Orange (ThermoFisher Scientific) prior to thermal unfolding to 96°C at a heating rate of 0.1 °C.sec^-1^ using a QuantStudio Flex 7 Real-Time PCR system (ThermoFisher Scientific). Fluorescence data were collected at Ex/Em 470/570 nm. Following subtraction of background fluorescence, the first derivative of the fluorescence data was determined and analyzed using GraphPad Prism (Version 8) to determine the *T_m_*. Data were collected from at least three biological replicates.

### Competitive Zn(II) Binding Experiments

Fluorescence of 150 nM Mag-Fura-2 (ThermoFisher Scientific) (Ex/Em 340/510 nm) saturated with Zn(II) was measured on a CLARIOstar Plus spectrophotometer (BMG Labtech) at 25°C in response to increasing concentrations of metal-free protein. Five technical replicates of each sample were analyzed in a black half-volume 384-well microtiter plate (Greiner Bio One). All experiments were performed in Chelex-100 treated 50mM MOPS (pH 7.2) 200 mM NaCl buffer. Fluorescence values were analyzed using a one-site non-linear fit model in GraphPad Prism (Version 8) using the experimentally-derived *K_D_* of Mag-Fura-2 (47 nM) to determine the *K_D_*for Zn(II) binding by AdcAII.

### X-Ray Crystallography and Structure Determination

Mutant AdcAII proteins were purified to homogeneity and concentrated to approximately 10 mg.mL^-1^ in centrifugal filter units (Amicon MWCO 30 kDa, Millipore). Crystals were obtained at 18 - 20°C using the hanging drop vapor diffusion technique in the following conditions: AdcAII_H65A_ and AdcAII_E280Q_, 0.1 M imidazole pH 8.0, 0.2 M zinc acetate, 20% (w/v) polyethylene glycol (PEG) 3350; AdcAII_H205L_, 0.2 M zinc acetate, 20% (w/v) PEG 3350. Crystals were mounted onto Cryoloops (Hampton Research) and briefly soaked in Paratone (Hampton Research) or 25%(v/v) glycerol prior to flash freezing in liquid nitrogen. X-ray diffraction data were collected at the Australian Synchrotron MX beamlines (McPhillips et al., 2002). Diffraction data were indexed and integrated using *XDS* (Kabsch, 2010) and scaled and merged using *Aimless* (Evans and Murshudov, 2013). Structures were determined using the molecular replacement technique in Phenix.Phaser (Adams et al., 2010) using the crystal structure of wild-type AdcAII (PDB accession code: 3CX3) (Loisel et al., 2008) as the initial search model. Initial models were built in *Phenix.AutoBuild* (Terwilliger et al., 2008). The structures were refined iteratively using *Phenix.Refine* (Afonine et al., 2012) and manually modified in *COOT* (Emsley et al., 2010).

### Molecular Dynamics Simulations

Chain A in the crystal structure of Zn(II)-bound AdcAII (PDB-ID: 3CX3) was used as the starting structure for the wild-type AdcAII simulations. All crystallographic waters and sodium were removed. Missing atoms in the side chains of residues Thr142, Glu308, Glu309 and Lys253 were modelled using the side chains of other residues of the same type. The missing loop formed by residues 129-141 was reconstructed using the loop-building tool in SwissPDB Viewer (Guex and Peitsch, 1997). In the crystal structure, Zn(II) is coordinated by His65-Nε2, His141-Nε2, His205-Nε2 and Glu280. Consequently, these His residues were modelled with a hydrogen atom on the ND1. All other His residues were protonated based on hydrogen bonding potential with surrounding residues.

For simulations of the AdcAII_H65A_, AdcAII_H205L_ and AdcAII_E280Q_ mutants, the crystal structures of Zn(II)-bound AdcAII_H65A_, AdcAII-_H205L_ and AdcAII_E280Q_, respectively, were used as starting structures. As before, missing atoms in the side chains or missing loops were reconstructed. The protonation state for all His residues was the same as in the simulations of wild-type AdcAII. For simulations of Zn(II)-free AdcAII and the equivalent mutants, the setup was identical except that the Zn(II) ion was removed from the crystal structure.

For all simulation systems, the protein was placed in a rectangular box, solvated with water molecules and Na^+^ ions were added to neutralize the charge on the protein. Additional Na^+^ and Cl^-^ ions were added to obtain a final ionic strength of 100 mM NaCl. The system was energy-minimized using a steepest descent algorithm, followed by a 5 ns NPT run, where the protein backbone atoms were position-restrained with a force constant of 500 kJ mol^-1^.nm^-2^. For the wild-type protein as well as the three mutants, 750-ns NPT production simulations of the Zn-bound and Zn-free system, respectively, were run in duplicate (i.e. 4 proteins × 2 systems (metal-bound and metal-free) × 2 simulations for a total of 16 simulations). All simulations were carried out using the GROMACS package version 5.0.1 (Abraham et al., 2015) in conjunction with the GROMOS 54a7 force field (Schmid et al., 2011) for protein and the simple point charge (SPC) model for water (Berendsen et al., 1981). Simulations were carried out under periodic boundary conditions with at least 1.5 nm between the protein and the box wall. Non-bonded interactions were described using a twin-range cut-off scheme with a 0.8 nm cut-off for short-range interactions and a 1.4 nm cut-off for long-range interactions. For long-range electrostatic interactions beyond 1.4 nm, a reaction field correction was applied, which was developed to be used for simulations with GROMACS and the GROMOS force field. A relative dielectric constant of ϵ = 78.5 was used. Covalent bonds were constrained using the SHAKE algorithm (Ryckaert et al., 1977), while the geometry of water molecules was constrained using the SETTLE algorithm (Miyamoto and Kollman, 1992). A Berendsen (Berendsen et al., 1984) thermostat with coupling constants of 0.1 ps was used to maintain the temperature close to the reference value of 25°C, while the Berendsen barostat with coupling constants of 0.5 ps was used for maintaining pressure close to 1 bar. Simulations were carried out using a 2-fs time step. Initial velocities were randomly assigned from Maxwellian distributions at 25°C. Configurations were saved every 500 ps for analysis. Analysis was carried out using GROMACS tools and the python library MDAnalysis (Michaud-Agrawal et al., 2011). Unless otherwise stated, only the last 250 ns of each trajectory was used for analysis and independent simulations for a given system were analyzed separately. All images were prepared using VMD (Humphrey et al., 1996). Cluster analysis was carried out using the algorithm of Daura et al. (Daura et al., 1999a; Daura et al., 1999b), implemented in GROMACS tools. A backbone neighbor RMSD cut-off of 2.5 Å was used. From each of the simulations, the most dominant conformation was used for comparison between metal-bound and metal-free states.

### Data Availability

The accession codes for the structures deposited in the Protein Data Bank are: 7LM5 (Zn^2+^-bound AdcAII_H65A_), 7LM6 (Zn^2+^-bound AdcA_H205L_), and 7LM7 (Zn^2+^-bound AdcA_E280Q_).

## Results

### AdcAII Is Highly Conserved in *S. pneumoniae*

The conservation of AdcAII was assessed by comparing the amino acid sequence of AdcAII (SPD_0888) across 20,020 *S. pneumoniae* genomes. This revealed that AdcAII had 99.97% prevalence and 99.25% conservation across all genomes, comparable to conservation and prevalence levels of the multilocus sequence typing genes *aroE*, *gdhA*, *gki*, *ddI*, and *xpt* (**Supplementary Table 1**). Non-redundant sequence conservation analysis showed that 295 of 306 residues in AdcAII were more than 95% conserved between the 20,016 strains carrying *adcAII* (**Supplementary Figure 1A**). The Zn(II)-coordinating residues were absolutely conserved (100%) in all strains, and only four residues were found to have less than 80% conservation (**Supplementary Figure 1B**). The high level of conservation maintained in AdcAII contrasts with other major surface proteins involved in virulence and disease, such as PspC and PspA, which despite sharing the same cellular localization, show significantly lower prevalence and conservation (**Supplementary Table 1**). These data suggest that mutations that perturb the Zn(II)-coordinating site or alter global protein structure are poorly tolerated in AdcAII. It therefore follows that the conservation of AdcAII and its Zn(II)-recruiting function are important for pneumococcal survival.

### Zinc Binding Induces Localized Structural Changes in AdcAII

We next sought to investigate the structure and Zn(II)-binding mechanism of AdcAII. First, we attempted to crystallize metal-free AdcAII, to determine the structure of the open, ligand-free state. Although crystals of metal-free AdcAII were obtained, they had a diffraction limit of 4 Å resolution, with poorly resolved electron density maps, and structure determination was not pursued further. Nevertheless, the domain-linking helix region (residues 168-194) could be built into the electron density map (data not shown). This suggested that in the absence of Zn(II) stabilization, there may be complexity within the conformational landscape of the protein. This may arise from the N- and C-terminal lobes, or discrete regions therein, exhibiting mobility/flexibility and/or adopting distinct conformational positions in the absence of the metal ion. We speculated that these regions within AdcAII could be restrained, and a partially open conformation achieved by mutating each of the four Zn(II)-coordinating residues: His65, His141, His205 and Glu280. For this, the Zn(II)-coordinating residues were substituted with an uncharged residue. Alanine was substituted for His65, which has recently been implicated as a component of a crucial mobile loop in *S. pneumoniae* AdcA (Luo et al., 2021), and His141. Leucine and glutamine were substituted for residues His205 and Glu280, respectively, to preserve steric bulk. Here, we report the crystal structures of the mutant variants in the Zn(II)-bound state: Zn-AdcAII_H65A_ (PDB: 7LM5), Zn-AdcAII_H205L_ (PDB: 7LM6), and Zn-AdcAII_E280Q_ (PDB: 7LM7). Although the AdcAII_H141A_ mutant variant was generated, it was refractory to structure determination. Detailed crystallography data statistics for the AdcAII variants are summarized in **Supplementary Table 2**.

**Figure 1:**
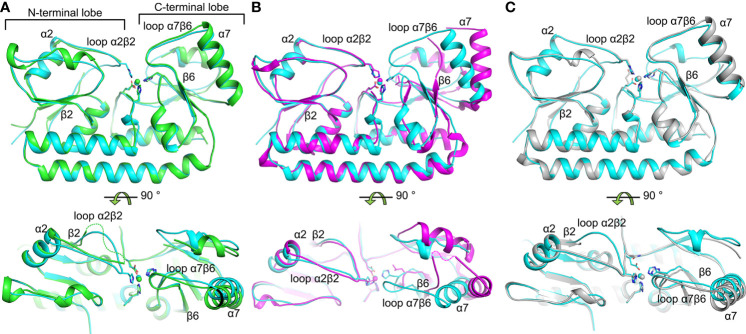
Structural comparisons of *S. pneumoniae* AdcAII wild-type and mutant variants. **(A–C)** depict the superposition of crystal structures of the wild-type Zn(II)-bound AdcAII (PDB ID: 3CX3, cyan) and AdcAII_H65A_ (PDB ID: 7LM5, green), AdcAII_H205L_ (PDB ID: 7LM6, magenta), and AdcAII_E280Q_ (PDB ID: 7LM7, grey), respectively. The bound Zn(II) ions are shown as spheres and their coordinating residue side chains as sticks. Missing loops in **(A, B)** are indicated by dotted lines in the bottom panels.

The AdcAII variant crystal structures all show a canonical cluster A-I SBP fold similar to the previously reported structure of wild-type Zn(II)-bound AdcAII [Zn-AdcAII; (Loisel et al., 2008)] (**Supplementary Figure 2**). Despite the absence of one of the metal-coordinating residues, each of the variants contained a single Zn(II) atom at the metal-binding site (**Supplementary Figure 3**). Coordination bond lengths of approximately 2.1 Å were observed in all structures, indicative of typical Zn(II)-protein binding. The Cα-Cα root-mean-square deviations (RMSDs) between Zn-AdcAII and Zn-AdcAII_H65A_, Zn-AdcAII_H205L_ and Zn-AdcAII_E280Q_ were 0.44, 0.66 and 0.47 Å, respectively, indicating no major deviation in main chain conformation. Nevertheless, significant differences were observed by comparison with Zn-AdcAII. Notably, Zn-AdcAII adopts a conformation wherein the metal-binding site is fully occluded from solvent, whereas Zn-AdcAII_H65A_ and Zn-AdcAII_H205L_ showed distinct surface conformations in the N- and C-terminal lobes, respectively. In the Zn-AdcAII_H65A_ structure, electron density for the α2β2 loop (residues 60-67) was not observed, suggesting that the loop is more mobile in the absence of Zn(II) and stabilized by Zn(II) coordination *via* His65 (**Figure 1A**), as was recently implicated in structural analysis of AdcA (Luo et al., 2021). Thus, mutation of His65 resulted in exposure of the bound Zn(II) ion to bulk solvent on the N-terminal side of the binding site. The structure of Zn-AdcAII_H205L_ also showed noticeable deviation from the Zn-AdcAII structure, with the differences primarily localized to the C-terminal lobe of the protein (**Figure 1B**). These manifested as rotation of helices α7 (32°) and α8 (28°); loss of H-bonding between β-strands β5 and β6; and disorder for part of loop α7β6 (residues 223-233), as evidenced by the lack of electron density in this region. These changes suggest that metal coordination by His205 facilitates formation of the β5-β6 interface, resulting in the movement of loop α7β6 and subsequent rotation of helices α7 and α8. Notably, as loop α7β6 shields the entrance of the metal-binding site, its absence exposes the bound Zn(II) to bulk solvent from the C-terminal lobe. Thus, loops α7β6 and α2β2 seem to serve as the gating loops of the metal-binding site, with Zn(II)-binding directly coupling to the closure of the metal binding site.

Finally, structure determination of Zn-AdcAII_E280Q_ revealed negligible global differences by comparison with Zn-AdcAII, with the metal-binding site and bound Zn(II) ion occluded from solvent (**Figure 1C**). The point mutation appeared to preclude Gln280 interaction with Zn(II), with the Oε1 atom ~4.4 Å distant from the metal. In wild-type AdcAII the Glu280 Oε2 atom contributed to the coordination of the metal (~2.3 Å from the Zn(II) ion) as the Oε1 atom was outside of the ideal range for interaction (~3.2 Å from the Zn(II) ion). Taken together, these data indicate that coordination of the Zn(II) by Glu280 does not appear to play a major role in the conformational changes. Thus, the role of residue Glu280 appears to be in completing the metal coordination sphere and reducing the solvent accessibility of the metal-binding site in the Zn(II)-bound state. Collectively, structural analyses of AdcAII variant proteins show that metal-binding by His65 and His205 is directly coupled to protein conformational changes. These were not observed for Glu280, while His141 remained refractory to this approach.

### Molecular Dynamics Simulations of AdcAII and Mutant Variants

**Figure 2:**
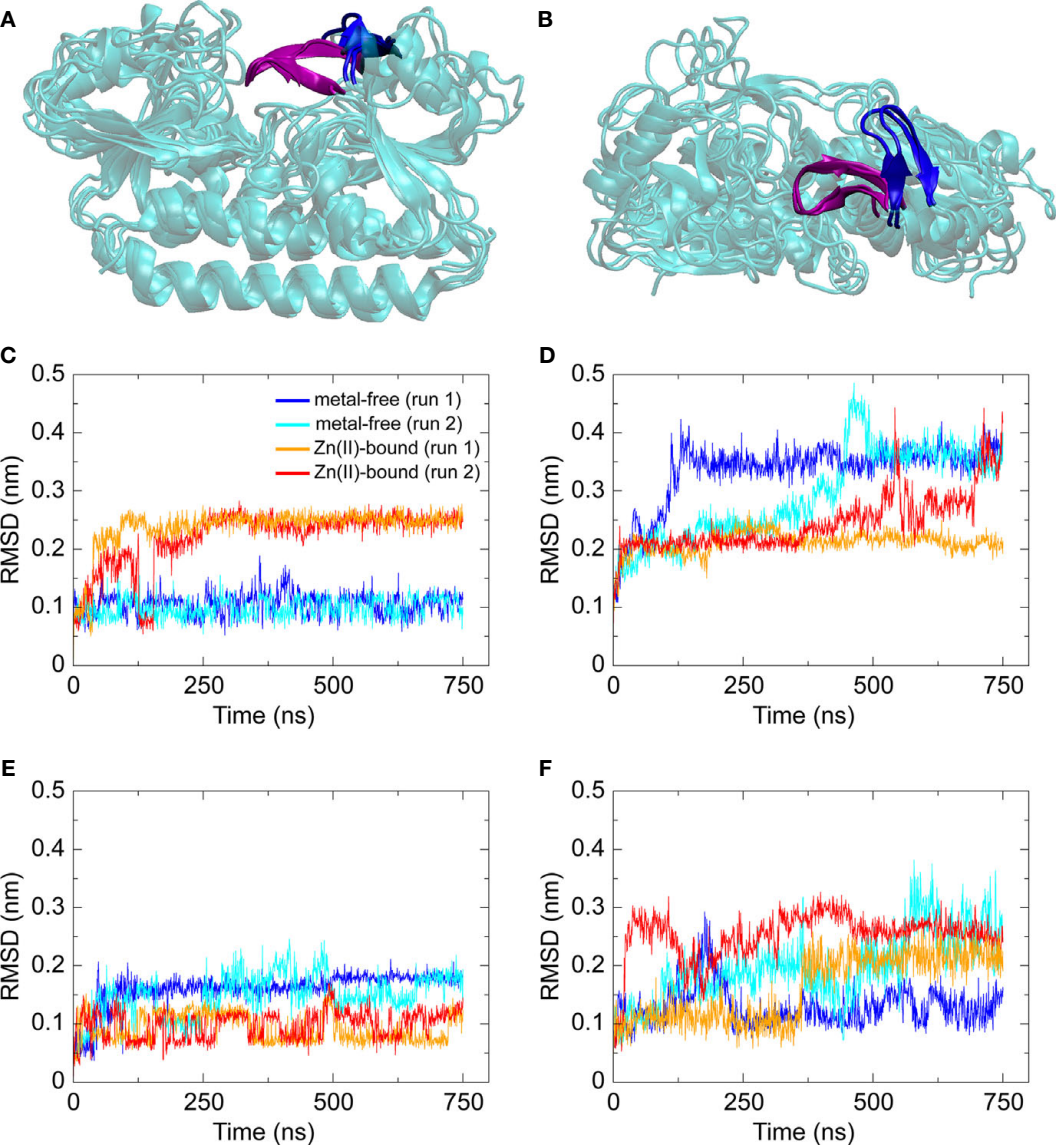
Structures and RMSD *vs.* time data from MD simulations of Zn(II)-bound and metal-free wild-type AdcAII. **(A, B)** Dominant conformations, as determined by clustering analysis, from duplicate simulations of metal-free and Zn(II)-bound AdcAII. The protein is shown in cartoon representation in cyan. Loop α2β2, which contains His65, is shown in purple for the Zn(II)-bound state and in blue for the metal-free state. **(C–F)** RMSD (nm) *vs*. time (ns) data for the four metal-coordinating loops – loop α2β2 containing His65 **(C)**, loop α3β4 containing His141 **(D)**, loop α6β5 containing His205 **(E)**, and loop α9β8 containing Glu280 **(F)** – calculated from duplicate (n = 2) 750 ns simulations of metal-free and Zn(II)-bound AdcAII. For all four plots, data from each simulation (two metal-free, two Zn(II)-bound) state are shown and color-coded as denoted in **(C)**.

Building on the structural insights into AdcAII and the mutant variants, molecular dynamics (MD) simulations were used to further study the SBP. First, MD simulations were performed on metal-free and Zn(II)-bound wild-type AdcAII. Simulations for the Zn(II)-bound wild-type AdcAII were set up using the corresponding crystal structure. Due to the lack of a metal-free structure, Zn(II)-bound AdcAII with the metal ion removed was used as a starting structure. Metal-free and Zn(II)-bound states of wild-type AdcAII were simulated for 750 ns in duplicate. For all analyses, the last 250 ns of the relevant trajectories were used. Cluster analysis on all backbone atoms with a cut-off = 2.5 Å was used to identify the dominant conformations sampled in the simulations of metal-free and Zn(II)-bound wild-type AdcAII. Comparison of these structures indicates that the global protein fold is not affected by the presence of Zn(II), suggesting that no large-scale structural rearrangements are triggered by AdcAII-metal binding. In addition, no significant differences in the root-mean square fluctuations (RMSF) between Zn(II)-bound and metal-free states were observed. This suggested that the overall mobility of different protein domains remains unchanged in the presence and absence of Zn(II) (**Supplementary Figure 4A**). To further assess conformational differences between the two states of the protein, the average pairwise Cα-Cα distances were calculated between all possible residue pairs over 250 conformations, for each respective simulation. The differences between each Cα-Cα distance in the Zn(II)-bound and metal-free state was then determined (Δ_Cα-Cα_ distance). The Δ_Cα-Cα_ distances provide insight into regions of the protein that adopt different conformations in the metal-free state relative to the Zn(II)-bound state of the protein. This revealed that loop α2β2, which contains the metal-binding site residue His65, adopted different relative positions in simulations of the two states (**Figures 2A, B**). Quantification of changes between the metal-free and Zn(II)-bound simulations was performed by RMSD *vs*. time analysis (**Figures 2C–F**). This revealed that only loop α2β2 showed a significant difference in comparison of the two states (99% confidence interval). The three loops that contain each of the other metal-coordinating residues – loop α3β4 (His141); loop α6β5 (His205); and loop α9β8 (Glu280) – showed much lower differences between the metal-free and Zn(II)-bound simulations (68% confidence interval). Analysis further showed that in the metal-free state, loop α2β2 moved away from its original position within the first ~100 ns of the simulation and remained in this same position for the rest of the simulation. On the other hand, loop α7β6, which was predicted to be stabilized upon Zn(II) coordination by His205 from the Zn-AdcAII_H205L_ crystal structure, showed a much less pronounced increase in mobility in the absence of Zn(II). This suggests that of the four loops that contain the metal-binding residues, loop α2β2 shows the most pronounced change in conformation upon Zn(II) binding.

In addition to the simulations of wild-type AdcAII, we performed MD simulations on the AdcAII variants AdcAII_H65A_, AdcAII_H205L_ and AdcAII_E280Q_ (**Supplementary Figures 4B, 5**). For the Zn(II)-bound simulations, the corresponding crystal structures were used. As performed for the wild-type, the metal-free systems were setup by removing the metal from the Zn(II)-bound structure. For all three variants, simulations of metal-free and Zn(II)-bound states were carried out for 750 ns, in duplicate, with the last 250 ns used for analysis. Analysis of the AdcAII_H205L_ and AdcAII_E280Q_ variants revealed no significant differences in residue position or mobility between the metal-free and Zn(II)-bound simulations, the latter of which is consistent with the minimal structural changes observed in the Zn-AdcAII_E280Q_ structure (**Supplementary Figure 5** **;** **Supplementary Tables 3, 4**). Simulations of AdcAII_H205L_ showed that changes in the C-terminal lobe were attributable to the untethering of His205 from the metal-binding site and the inability to form the β5-β6 interface. An additional 750 ns simulation was conducted on Zn(II)-bound AdcAII_H205L_ with analyses revealing that, in two (of the three) independent simulations, Zn(II) moves out of its solvent-exposed metal binding site. Thus, the MD simulations support inferences from the crystal structure that the role of loop α7β6 is to reduce bulk solvent access to the metal-binding site mediated *via* Zn(II)-coordination with His205.

**Figure 3:**
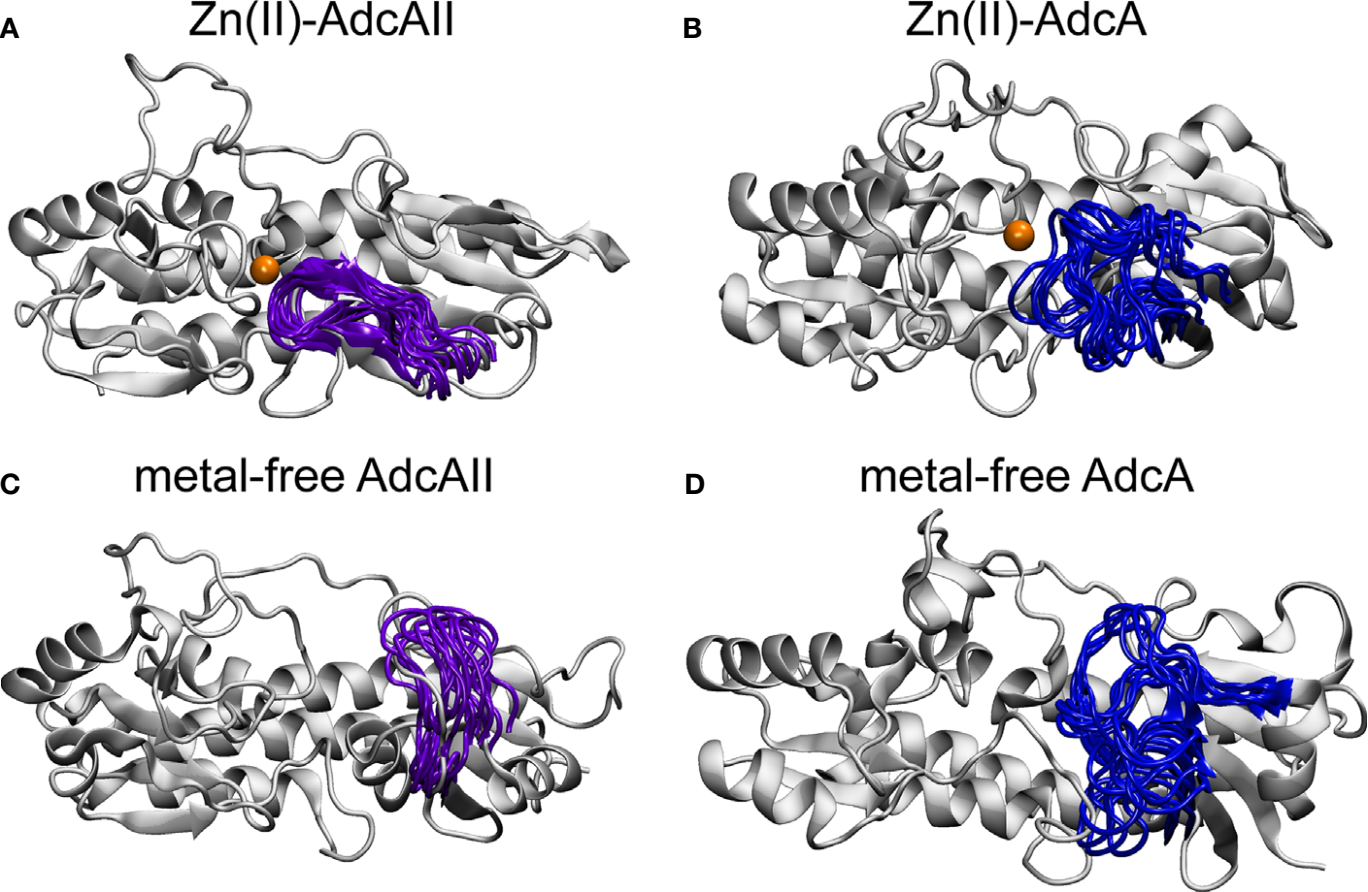
Comparison of α2β2 loop in AdcAII and the AdcA N-terminal cluster A-I domain. Conformation of α2β2 loop in AdcAII **(A, C)** and AdcA **(B, D)**, in the Zn(II)-bound **(A, B)** and metal-free **(C, D)** states. The protein is shown in cartoon representation in grey with the α2β2 loop shown in purple (AdcAII) and dark blue (AdcA). The Zn(II) ion is shown in orange. Conformations for the movement of the α2β2 loop are taken from conformations of duplicate 750-ns simulations of the AdcAII and A-I AdcA domain.

Simulations of AdcAII_H65A_ revealed that in the metal-free state, loop α2β2 and helix α2 adopt unique conformations that are not observed in the Zn(II)-bound state of the AdcAII_H65A_ variant or in either state of wild-type AdcAII (**Supplementary Figure 4B**). As noted above, the Zn-AdcAII_H65A_ crystal structure did not show electron density for the α2β2 loop (residues 60-67), which indicates this loop has greater mobility in the variant protein than the wild-type. However, this does not imply that α2β2 loop is disordered as the lack of electron density can arise from the loop adopting multiple distinct conformations. While these conformations would be refractory to crystallographic approaches, they can be captured by the MD simulations. When considered in context with the Zn-AdcAII_H65A_ structure, these data suggest that loop α2β2 controls access to the N-terminal side of the metal-binding site, with its conformation being restricted by interaction of His65 with the Zn(II) ion. Intriguingly, the loop α2β2 region was recently shown to be critical for the function of AdcA for *S. pneumoniae* Zn(II) uptake in a Δ*adcAII* background (Luo et al., 2021). However, the high mobility and dynamic conformational sampling of loop α2β2 in the metal-free state of AdcA is distinctly different from the equivalent region in AdcAII (**Figure 3**). In AdcA, loop α2β2 adopts different conformations in the two states of the protein, with the region demonstrating high mobility in the absence of the Zn(II). By contrast, although loop α2β2 adopts different conformations in the Zn(II)-bound and metal-free states of AdcAII, the loop does not exhibit dynamic mobility. This suggests that despite sharing similar global architectures, there are subtle conformation and mechanistic differences in the Zn(II)-binding mechanism of these two SBPs. Collectively, our biophysical analyses of AdcAII and its binding-site mutant derivatives strongly suggest that loops α2β2 and α7β6 are involved in conformational changes upon Zn(II) binding.

### Metal-Binding Properties of Wild-Type AdcAII and Mutant Variants

**Figure 4:**
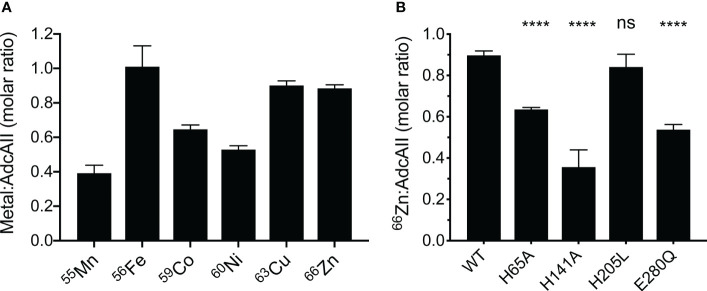
Biochemical analyses of recombinant wild-type AdcAII and mutant variants. **(A)** *In vitro* metal-binding experiments of metal-free AdcAII with first-row transition metal ions are shown. Data represent the mean molar ratio of metal to AdcAII (± SEM) determined by ICP-MS from at least three independent biological experiments. **(B)** *In vitro* Zn(II)-binding experiments of AdcAII mutant variants AdcAII_H65A_, AdcAII_H141A_, AdcAII_H205L_ and AdcAII_E280Q_ analyzed by ICP-MS. Data represent the mean molar ratio of metal to protein (± SEM) from at least three independent biological experiments. Statistical analyses comparing mutant variants to the wild-type protein were performed by one-way ANOVA with Tukey post-test with *P*-values of > 0.05 and < 0.0001 are denoted by ns and ****, respectively.

To further probe the role of each Zn(II)-coordinating residue in AdcAII, we examined the metal-binding properties of the protein using *in vitro* metal-loading experiments, differential scanning fluorimetry (DSF), and affinity determination assays. *In vitro* metal-loading of AdcAII assessed binding of metal ions by inductively coupled plasma-mass spectrometry (ICP-MS). Wild-type AdcAII bound 0.89 ± 0.07 Zn(II) atoms per molecule, consistent with a single metal-binding site (Loisel et al., 2008). AdcAII was also shown to be permissive for interaction with the first-row transition metal ions Mn(II), Co(II), Ni(II) and Cu(II), albeit to varying extents (**Figure 4A**). Variation in the extent of the metal-bound states can most likely be attributed to cation dissociation due to sample washing prior to ICP-MS analysis. The metal-binding behavior of AdcAII was in stark contrast to AdcA, which is highly specific for Zn(II) and does not appreciably interact with other divalent cations (Plumptre et al., 2014; Luo et al., 2021). We then examined the *in vitro* Zn(II)-binding of the AdcAII mutant variants, to understand the impact of the mutations on interaction with the physiological ligand. *In vitro* binding of Zn(II) was severely impaired in AdcAII_H141A_, significantly reduced in AdcAII_H65A_ and AdcAII_E280Q_, but not significantly affected in AdcAII_H205L_ (**Figure 4B** **)**. Collectively, these data demonstrate that AdcAII is not restricted to interacting solely with Zn(II), despite extensive evidence (Loisel et al., 2008; Bayle et al., 2011; Bersch et al., 2013; Plumptre et al., 2014; Brown et al., 2016) indicating that it is the primary physiological ligand. Further, mutation of the metal binding site residues impacts Zn(II) binding, albeit to different extents.

We then analyzed wild-type AdcAII and the mutant variants by DSF, to ascertain how metal-protein interactions influenced SBP thermostability. In wild-type AdcAII, Zn(II) and Co(II) induced significant melting temperature (*T_m_*) shifts, Mn(II), Ni(II), and Cu(II) induced smaller, but still significant, *T_m_* increases, while Fe(II) did not significantly alter the *T_m_* (**Table 1**). These data suggest that the metal-binding site of AdcAII can interact to some degree with the majority of first row transition metal ions, consistent with the ICP-MS results. Nevertheless, the thermostability shifts observed were relatively small for cations other than Co(II) or Zn(II). We speculate that differences in coordination of the non-cognate cations at the AdcAII metal-binding site is most likely attributable for the variations in observed thermostabilities of protein-metal complexes. Whether the interaction with other cations can facilitate productive import is unclear. To date, the contribution, if any, of the AdcCB importer to regulating metal import remains undefined. Future studies to address this question would also need to consider the contribution of the *S. pneumoniae* metal export systems MntE, CopA, and CzcD.

**Table 1:** Effect of transition metal ions on the melting temperature of AdcAII.

Treatment	*T_m_* (˚C)^a^	Δ*T_m_*
Metal-free	51.01 ± 0.16	–
Mn(II)	54.96 ± 0.34^b^	+3.95
Fe(II)	53.09 ± 0.24	+2.20
Co(II)	70.08 ± 2.19^b^	+19.08
Ni(II)	57.15 ± 0.24^b^	+6.14
Cu(II)	60.48 ± 0.66^b^	+9.38
Zn(II)	75.92 ± 0.57^b^	+24.91

a. Values shown represent the average and standard error of the mean from at least 3 independent measurements.

b. Statistically significant difference to metal-free protein T_m_ (one-way ANOVA with Tukey post-test).

DSF analyses of the AdcAII mutant variants revealed that the binding site mutations had no effect on the thermostability of the proteins in the metal-free state, by comparison with the wild-type protein (**Table 2**). This indicates that the binding site mutations do not destabilize the global protein structure of the SBPs. DSF analysis of the AdcAII variants with the cognate ligand showed a significant reduction in the Δ*T_m_* of all variants (**Table 2**). Zn(II)-AdcAII_H141A_ showed the lowest Δ*T_m_*, with a reduction of 18.75 °C relative to wild-type protein. This was followed by Zn(II)-AdcAII_H65A_ and Zn(II)-AdcAII_H205L_, which showed similar Δ*T_m_* reductions of 16.56 °C and 13.16 °C, respectively, by comparison with wild-type AdcAII, while in Zn(II)-AdcAII_E280Q_, the Δ*T_m_* was reduced by only 9.91°C relative to the wild-type protein. In summary, the DSF analyses show that all four of the metal-coordinating residues contribute to ligand-induced stabilization of AdcAII. Accordingly, we sought to further investigate the impact of the binding site mutations by determining the affinity of wild-type AdcAII and the variants for Zn(II).

**Table 2:** Effect of zinc on the melting temperature of AdcAII binding site variants.

	Metal-free *T_m_* (˚C) ^a^	Zn(II) *T_m_* (˚C)	Δ*T_m_*(˚C)
WT AdcAII	51.01 ± 0.16	75.92 ± 0.57^b^	+24.91
H65A	51.52 ± 0.61	59.86 ± 0.78^b^	+8.35^c^
H141A	49.11 ± 0.69	54.99 ± 0.87^b^	+6.16^c^
H205L	49.90 ± 0.37	61.65 ± 0.64 ^b^	+11.75^c^
E280Q	50.31 ± 0.54	65.31 ± 1.02^b^	+15.00^c^

a. Values shown represent the average and standard error of the mean from at least 3 independent measurements.

b. Statistically significant difference to metal-free protein T_m_ (one-way ANOVA with Tukey post-test).

c. Statistically significant difference to WT AdcAII ΔT_m_ (unpaired t-test, P < 0.0001).

The dissociation constant (*K_D_*) of AdcAII for Zn(II) was determined using a competitive binding assay with the metal-responsive fluorophore MagFura-2. Wild-type AdcAII bound Zn(II) with an experimentally derived *K_D_* of 18.7 ± 2.1 nM (**Table 3** and **Supplementary Figure 6**) and is within the sub-nanomolar to micromolar range of previously characterized Zn(II)-specific cluster A-I SBPs (Desrosiers et al., 2007; Wei et al., 2007; Lim et al., 2008; Plumptre et al., 2014; Handali et al., 2015). Affinity determination for the AdcAII variants revealed that all mutants had reduced affinity for Zn(II) relative to wild-type AdcAII and followed the order of: AdcAII > AdcAII_H205L_ > AdcAII_E280Q_ > AdcAII_H65A_ > AdcAII_H141A_ (**Table 3** and **Supplementary Figure 6**). These data show that mutation of His141 compromised interaction of AdcAII with Zn(II) to the greatest extent, consistent with the refractory nature of the mutant SBP to structure determination. Mutation of either Glu280 or His65 resulted in mutants compromised to similar extents with respect to interaction with Zn(II). Intriguingly, although mutation of His205 reduced the thermostability of the SBP-metal complex and resulted in displacement of the C-terminal lobe of the protein, the AdcAII_H205L_ variant showed higher affinity for Zn(II) and a greater capacity to retain the bound ion compared with the other mutants. Taken together, these data suggest that AdcAII_H205L_ is capable of efficient Zn(II) recruitment but would likely be compromised for efficacious uptake due to the inability of the SBP to transition to a closed conformation. Accordingly, we investigated how the binding site mutations affected Zn(II) acquisition by the Adc permease in *S. pneumoniae*.

**Table 3:** Effect of metal binding site mutations on *in vitro* Zn(II) binding.

AdcAII variant	Dissociation Constant *K_D_* (nM)
WT AdcAII	18.7 ± 2.1
AdcAII_H65A_	174.4 ± 30.7
AdcAII_H141A_	247.8 ± 63.8
AdcAII_H205L_	44.7 ± 3.6
AdcAII_E280Q_	159.3 ± 42.7

### Conformational Changes in AdcAII Are Required for Efficacious Zinc Uptake

**Figure 5:**
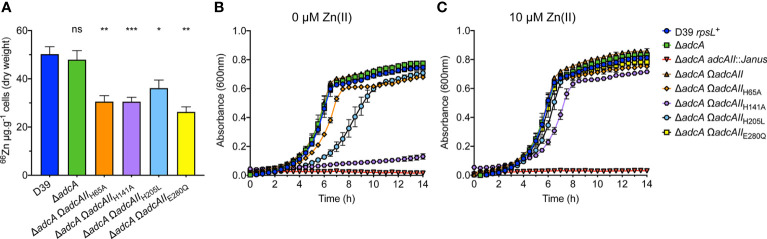
Phenotypic effects of binding site mutations in AdcAII. **(A)** Intracellular Zn(II) accumulation of *S. pneumoniae* D39 and *adcAII* mutant strains in C+Y media supplemented with 10 µM ZnSO_4_ as determined by ICP-MS. Data correspond to mean (± SEM) µg Zn.g^-1^ cell measurements from at least three independent biological experiments. Statistical significance of the differences in concentrations of mutant variants compared to the wild-type strain were determined by a one-way ANOVA with Dunnett post-test. *P*-values of >0.05, <0.05, <0.005 and <0.0005 are denoted by ns, *, ** or ***, respectively. **(B)** *In vitro* growth measurements of wild-type *S. pneumoniae* D39 and *adc* mutant strains. Bacteria were grown in CDM [~0.2 µM Zn(II)], incubated at 37°C, 5% CO_2_ and growth monitored by OD_600_ at 30 min intervals. **(C)** Bacteria were grown in CDM supplemented with 10 µM Zn(II), incubated at 37°C, 5% CO_2_ and growth monitored by OD_600_ measurements at 30 min intervals. Data for **(B, C)** are representative mean (± SEM) from at least three independent biological experiments.

The impact of the binding site mutations on pneumococcal Zn(II) uptake was addressed by generating mutant *S. pneumoniae* strains in the D39 Δ*adcA* background (Plumptre et al., 2014), which excluded the contribution of AdcA to Zn(II) import (**Supplementary Table 5**). Growth kinetics and zinc uptake in the *S. pneumoniae adcAII* mutant strains were then assessed in cation-defined minimal media (CDM), while whole cell metal accumulation studies used Zn(II)-replete (10 µM) C+Y medium. This latter variation was necessitated by the poor biomass recovery of CDM grown cultures for whole cell metal accumulation analyses, which was overcome by use of C+Y growth medium. The *S. pneumoniae* wild-type and Δ*adcA* strain showed similar levels of Zn(II) accumulation, consistent with prior studies (**Figure 5A**) (Plumptre et al., 2014). Although the *S. pneumoniae adcA adcAII* double knockout strain (Δ*adcA adcAII*::*Janus*) grew, it failed to generate sufficient biomass for metal content determination. The *S. pneumoniae* strains encoding the *adcAII* binding site mutants revealed that all strains had significantly reduced Zn(II) accumulation relative to the parental strain (**Figure 5A**). However, there was no significant difference in Zn(II) accumulation between the individual mutant strains. This indicated that all strains acquired sufficient Zn(II) for viability in Zn(II)-replete C+Y, but there was insufficient resolution using this growth medium to assess any differences between the strains. To further investigate differences between strains, we employed Zn(II)-depleted CDM that contained less than 200 nM Zn(II).

The *S. pneumoniae* wild-type and Δ*adcA* strain showed similar growth phenotypes in CDM, while the double mutant, Δ*adcA adcAII*::*Janus*, was greatly attenuated (**Figure 5B**). The *S. pneumoniae* Δ*adcA* AdcAII_H141A_ complemented strain (Δ*adcA* Ω*adcAII*_H141A_) demonstrated very poor growth in CDM, consistent with the impact of the mutation observed in our other analyses. The Δ*adcA* Ω*adcAII*_H65A_ strain showed a minor defect in growth, while the growth phenotype of the Δ*adcA* Ω*adcAII*_E280Q_ strain was comparable to the wild-type and parental strains. These data indicate that the impact of the binding site mutations on AdcAII Zn(II) affinity only manifests itself as a modest effect on bacterial Zn(II) uptake and growth in Zn(II)-restricted conditions. Intriguingly, the growth phenotype of the Δ*adcA* Ω*adcAII*_H205L_ strain was perturbed to a greater extent than the strains harboring either the His65 or Glu280 mutations. All *S. pneumoniae* binding site mutant strains demonstrated a growth phenotype comparable to the wild-type strain when supplemented with 10 µM Zn(II) (**Figure 5C**). These data show that despite the AdcAII_H205L_ variant retaining a higher affinity for Zn(II), this is not sufficient for optimal growth in Zn(II)-restricted conditions. It therefore follows that the inability of the AdcAII_H205L_ variant to support optimal *S. pneumoniae* growth is due to another impact of the mutation. The AdcAII_H205L_ structure shows that the mutant variant is unable to transition to a closed conformation with the C-terminal lobe essentially ‘untethered’ from the metal-binding site. Therefore, we speculate that the inability to adopt the metal-bound, closed conformation, impairs Zn(II) uptake in the Δ*adcA* Ω*adcAII*_H205L_ strain. However, the dynamics of Zn(II) release from AdcAII, how this is affected in mutant variants, and the impact of the mutation(s) on AdcB-AdcAII interaction likely also contribute and warrant further investigation to comprehensively understand the mechanisms of Zn(II) uptake *via* the Adc permease.

### Proposed Zn(II)-Binding Mechanism of AdcAII

Our analyses of AdcAII reveal that the protein does not undergo global conformational changes and that the localized structural rearrangements are also distinct from the mechanisms reported for other cluster A-I SBPs, notably the “spring-hammer” [PsaA (Couñago et al., 2014)], “partial domain slippage” [ZnuA (Chandra et al., 2007)] and the recently reported “trap-door” of AdcA (Luo et al., 2021). The data show that Zn(II)-binding in AdcAII is accompanied by localized structural rearrangements in the C-terminal lobe and stabilization of two loops that close the metal binding site from bulk solvent. Residue His65 appears to transition in and out of the coordination sphere, while His205 drives conformational changes in the C-terminal lobe, and Glu280 remains relatively static. Although the contribution of residue His141 to conformational changes remains unclear, it is unlikely to exhibit movement by virtue of its location deep within the binding pocket. Thus, residues His141 and Glu280 likely comprise the relatively pre-formed metal-binding site, with His65 and His205 playing a more dynamic role in Zn(II) binding. The interaction of His65 with Zn(II) facilitates movement of loop α2β2 onto the binding site. Interestingly, although loop α2β2 is mobile in AdcAII, it lacks the extensive mobility observed for the comparable loop in AdcA (Luo et al., 2021). This highlights that loop α2β2 contributes differently to the Zn(II) recruitment process in the two SBPs, despite their interaction with the same transporter. Conformational changes in the C-terminal lobe are driven by residue His205 in loop α6β5, which directly pulls β5 into a β-sheet with β6, causing β6 to tilt down and allowing loop α7β6 to close over the metal-binding site. Consequently, helices α7 and α8 are also pulled towards the metal-binding site. In the absence of Zn(II), the hydrogen bonds between β5 and β6 are largely not formed, and loop α7β6 is relaxed and moves away from the binding site. Stabilization of these structural elements in the C-terminal lobe may contribute to interaction of AdcAII with AdcB, although further studies will be required to investigate SBP-transporter interaction and the dynamics of Zn(II) release and import. In summary, AdcAII undergoes localized structural rearrangements upon Zn(II) coordination by two of the four coordinating residues, whereby the stabilization of flexible loops and the C-terminal lobe facilitates the transition to a closed conformation.

## Discussion

*S. pneumoniae* survival is dependent on the efficacious acquisition of Zn(II) from the host during infection (Bayle et al., 2011; Plumptre et al., 2014), with prior studies establishing the crucial role of AdcAII in initial infection and invasive disease (Plumptre et al., 2014; Brown et al., 2016). Here, we have shown that AdcAII is highly conserved at the amino acid sequence level in *S. pneumoniae* strains. This suggests that, distinct from other major surface proteins associated with virulence and disease such as PspA (Rapola et al., 2000) and PspC (Georgieva et al., 2018), AdcAII is not influenced by the selective pressures that drive antigenic variation. Whether this is due to poor immunogenicity of AdcAII limiting an antibody-mediated immune response, the antibody-targeted epitopes being shielded in some manner from surveillance, or a slow rate of evolution due to mutations not being tolerated in *adcAII*, remains to be determined. This work highlights that while mutations of the binding site do impact protein function, they do not necessarily compromise bacterial viability and growth in an *in vitro* context. This raises the question as to whether affinity for Zn(II) is important in absolute terms. The high affinity observed in AdcAII may have arisen as an evolutionary by-product in achieving an optimal conformation transition from the open, metal-free state to the closed, metal-bound closed state of the SBP. Simply put, the necessity of the SBP to be able to efficiently close upon Zn(II)-binding in order to productively interact with AdcCB transporter and thereby facilitate Zn(II) uptake, may have been the dominant selective pressure. The absolute affinity for Zn(II) ions, whether sub-nanomolar or micromolar, the range observed in other Zn(II)-specific SBPs, may not be crucial. Further studies of Zn(II)-specific systems and their relevant niches may provide further insight into this speculation.

The molecular and structural characterization of the AdcAII binding site mutants provides insight into the Zn(II) recruitment mechanism of the protein. Similar to other Zn(II)-specific SBPs, the metal-binding mechanism in AdcAII arises from localized conformational rearrangements within the SBP. The structural rearrangements in the C-terminal lobe of AdcAII have been observed in *E. coli* ZnuA (PDB: 2PS0 [Zn(II)-bound]; 2PS3 [metal-free]), suggesting that β-sheet formation and subsequent rotation of the α-helices are common features of Zn(II) SBPs (Yatsunyk et al., 2008). Structural studies of ZnuA, as well as *P. denitrificans* AztC (PDB: 5W57 and 5W56), *Candidatus* Liberibacter asiaticus ZnuA2 (PDB: 4UDN and 4UDO) and *S. pneumoniae* AdcA (PDB: 7JJ9 [AdcA] and 7JJ8 [N-terminal AdcA domain]) also show that the N-terminal lobe binding site His residue and the loop in which it resides, His65 and loop α2β2 in AdcAII, are commonly engaged in structural rearrangements upon Zn(II)-binding (Yatsunyk et al., 2008; Sharma et al., 2015; Neupane et al., 2017; Luo et al., 2021). However, our study reveals that although the Zn(II)-induced conformational changes are relatively subtle, their concerted action is required for high ligand affinity and optimal uptake of Zn(II). Preservation of these structural elements, notably loops α2β2 and α7β6 and the associated regions that facilitate their localized rearrangements, may be the underlying basis for the high degree of sequence conservation of *adcAII* across *S. pneumoniae* strains. Unexpectedly, the Zn(II)-binding mechanism of AdcA and AdcAII are subtly different despite both SBPs recruiting the same metal ligand and releasing it to the same transporter, AdcCB. In AdcA, the dynamic mobility of loop α2β2 has a crucial role in bacterial Zn(II) uptake. By contrast, although AdcAII loop α2β2 and His65 undergo a conformational transition during Zn(II)-binding, this loop region lacks the flexibility observed in AdcA and is less crucial in Zn(II)-binding and bacterial Zn(II) uptake. Further, loop α7β6 in the C-terminal lobe appears to be static in AdcA, whereas this region undergoes a conformational transition in AdcAII during Zn(II)-binding. Thus, these data suggest that metal-free AdcAII likely samples a more open conformation than metal-free AdcA.

The increased solvent accessibility and less rigidly defined metal-binding site in AdcAII provides a plausible explanation for the greater promiscuity of cation interaction. Although Zn(II)-bound AdcAII has near-identical coordination geometry to AdcA, the latter protein is restricted to only binding Zn(II), while AdcAII can interact with a broad range of divalent cations similar to *E. coli* ZnuA (Yatsunyk et al., 2008), *S. pneumoniae* PsaA (Couñago et al., 2014), and *Candidatus* ZnuA2 (Sharma et al., 2015). This has been attributed to the inherent flexibility of the amino acid side chains that comprise the metal-binding site in these SBPs, resulting in an inability to exclude cations other than Zn(II) from interacting and binding. The *in vitro* metal-binding and DSF data suggest that AdcAII is able to interact and potentially transition to a metal-bound, closed conformation with the non-cognate metal ions Co(II) and Cu(II). However, in the nasopharynx, the primary physiological niche colonized by *S. pneumoniae*, AdcAII is unlikely to encounter appreciable levels of these metal ions. Both Co(II) and Cu(II) are relatively low abundance elements in nasopharyngeal tissue, with Co(II) also frequently occurring as a cobalamin chelate in biological systems (Eijkelkamp et al., 2019). Thus, the promiscuity of AdcAII is unlikely to influence asymptomatic colonization of the pathogen. However, it may be a factor during invasive disease, where Cu(II) abundance can increase significantly (Eijkelkamp et al., 2019).

Unexpectedly, affinity for Zn(II), *in vitro* Zn(II)-binding, thermostability of the AdcAII-Zn(II) complex, and impact on bacterial growth were not congruently aligned for the mutant variants. It is notable that AdcAII_H205L_ retained *in vitro* Zn(II)-binding and affinity for Zn(II) to the greatest extent but was heavily compromised in the DSF assay and in supporting bacterial growth in Zn(II)-limited medium. By contrast, AdcAII_E280Q_ supported wild-type bacterial growth and was the least perturbed variant in the DSF assays but showed reduced *in vitro* Zn(II)-binding and affinity for Zn(II). It is tempting to speculate that the relative degree of conformational impact revealed by the mutant variant crystal structures is the dominant determinant for the observed functional impact. In isolation, this inference provides a plausible explanation for the observed effect on bacterial Zn(II) uptake in the *adcAII* mutant strains. However, while consistent with the data presented herein, this conclusion does not account for the contribution of AdcAII conformational dynamics (in the metal-free and metal-bound states) and interaction of protein-metal complexes with the AdcCB transporter, which remain to be determined. Further, it is important to note that there are energy barriers traversed during the metal-binding process that cannot be addressed by MD simulations using metal-free SBP states generated *via* the *in silico* removal of the Zn(II) ion from a closed, metal-bound structure. This latter issue is an unavoidable technical limitation arising from the absence of a metal-free crystal structure for AdcAII. Despite these caveats, our data suggest that the transition to a closed conformation contributes to efficacious bacterial Zn(II) uptake.

In addition to its metal ion selectivity and mechanistic differences, AdcAII also differs from AdcA in terms of accessory elements used for Zn(II) recruitment. Notably, AdcAII lacks the His-rich loop present in most Zn(II)-specific SBPs, which has recently been shown to aid in sampling bulk solvent for Zn(II) ions in AdcA (Luo et al., 2021) and in other Zn(II)-specific SBPs (Wei et al., 2007; Falconi et al., 2011; Neupane et al., 2017). AdcAII also lacks the C-terminal extension of AdcA that encodes a polypeptide with similarity to the ZinT protein of Gram-negative bacteria. In AdcA, this C-terminal domain also aids in Zn(II) recruitment (Plumptre et al., 2014; Luo et al., 2021). Despite lacking these structural features, AdcAII has the capacity to interact with four capsule-embedded Pht proteins, PhtABDE, to aid in Zn(II) uptake (Plumptre et al., 2014; Luo et al., 2021). *In vitro* biophysical studies have suggested direct interaction between AdcAII and PhtD, but definitive *in vivo* interaction studies remain to be performed (Loisel et al., 2011; Bersch et al., 2013). Thus, although the mechanistic basis for how AdcAII and the Pht proteins interact remains unclear, these two protein systems cooperatively support efficacious recruitment of Zn(II) in restricted nutritional conditions.

In conclusion, this work presents new insight into the structural and biochemical aspects of AdcAII. This study shows further variation on the mechanistic pathways to recruit Zn(II) ions by a cluster A-I SBP within a common protein architecture. This is particularly notable in *S. pneumoniae*, as despite the differences in Zn(II) recruitment, both AdcA and AdcAII facilitate transport of the bound metal *via* the ABC transporter, AdcCB. This work also suggests that SBP conformation may be more crucial than affinity in terms of Zn(II) acquisition, although *in vivo* experiments remain to be performed. Collectively, these structural and functional insights into AdcAII provide new mechanistic knowledge of the SBP that will be essential for the development of antimicrobial therapeutics targeting the pneumococcal Zn(II) acquisition pathways at the host-pathogen interface.

## Data Availability Statement

The datasets presented in this study can be found in online repositories. The names of the repository/repositories and accession number(s) can be found below: http://www.wwpdb.org/, 7LM5; http://www.wwpdb.org/, and 7LM6; http://www.wwpdb.org/, 7LM7.

## Author Contributions

MŽ, ED, BK, and CM contributed to conception and design of the study. MŽ and VP performed the microbiological assays. ZL performed the structural studies. ED performed the molecular dynamics studies. KG performed the ICP-MS. SN contributed strains, constructs, and supervised MŽ. MŽ wrote the first draft of the manuscript. ZL and ED wrote sections of the manuscript. MŽ and CM revised and integrated the manuscript text. All authors contributed to the article and approved the submitted version.

## Funding

This research was undertaken with the assistance of resources and services from the National Computational Infrastructure (NCI), which is supported by the Australian Government and by resources provided by The Pawsey Supercomputing Centre with funding from the Australian Government and the Government of Western Australia. This research was also undertaken using the LIEF HPC-GPGPU Facility hosted at the University of Melbourne. This Facility was established with the assistance of Australian Research Council (ARC) LIEF Grant LE170100200. This work was supported by the National Health and Medical Research Council (NHMRC) grants 1071659 to BK, 1122582 to CM and 1180826 to BK and CM. This work was also supported by the ARC Discovery Project Grant to CM (DP170102102). SN is an NHMRC Early Career Research Fellows (1142695), ED is a UTS Chancellor’s Postdoctoral Fellow, BK is an ARC Laureate Fellow (FL180100109), and CM is an ARC Future Fellow (FT170100006).

## Author Disclaimer

The content of this study is solely the responsibility of the authors and does not necessarily represent the official views of the funding bodies.

## Conflict of Interest

The authors declare that the research was conducted in the absence of any commercial or financial relationships that could be construed as a potential conflict of interest.

## Publisher’s Note

All claims expressed in this article are solely those of the authors and do not necessarily represent those of their affiliated organizations, or those of the publisher, the editors and the reviewers. Any product that may be evaluated in this article, or claim that may be made by its manufacturer, is not guaranteed or endorsed by the publisher.
