# Supplementary material for: Conformation of the Solute-Binding Protein AdcAII Influences Zinc Uptake in Streptococcus pneumoniae
Source: Front Cell Infect Microbiol. 2021 Aug 19;11:729981. doi: 10.3389/fcimb.2021.729981 (PMC8416893; doi:10.3389/fcimb.2021.729981)
Supplement: Supplementary file 1 [file DataSheet_1.pdf]

Supplementary Information

Conformation of the solute-binding protein AdcAII influences zinc uptake in *Streptococcus pneumoniae*

Marina L. Župan [1], Zhenyao Luo [2,3,4], Katherine Ganio [1], Victoria G. Pederick [5], Stephanie L. Neville [1], Evelyne Deplazes [6,7], Boštjan Kobe [2,3,4†], Christopher A. McDevitt [1†*].

1. Department of Microbiology and Immunology, The Peter Doherty Institute for Infection and Immunity, University of Melbourne, Melbourne, Victoria, Australia.
2. School of Chemistry and Molecular Biosciences, The University of Queensland, Brisbane, Queensland, Australia.
3. Australian Infectious Diseases Research Centre, The University of Queensland, Brisbane, Queensland, Australia.
4. Institute for Molecular Bioscience, The University of Queensland, Brisbane, Queensland, Australia.
5. Department of Molecular and Biomedical Science, School of Biological Sciences, The University of Adelaide, Adelaide, South Australia, Australia.
6. School of Life Sciences, University of Technology Sydney, Ultimo, New South Wales, Australia.
7. School of Pharmacy and Biomedical Sciences, Curtin Health Innovation Research Institute, Curtin University, Bentley, Western Australia, Australia.

† These authors share last authorship.

* Correspondence address: Christopher A. McDevitt, Department of Microbiology and Immunology, The Peter Doherty Institute for Infection and Immunity, The University of Melbourne, Melbourne, Victoria, 3000, Australia. Phone: 61-3-8344-7200. E-mail: christopher.mcdevitt@unimelb.edu.au

This file contains **Supplementary Figures 1-6** and **Supplementary Tables 1-7**.

SUPPLEMENTARY FIGURES

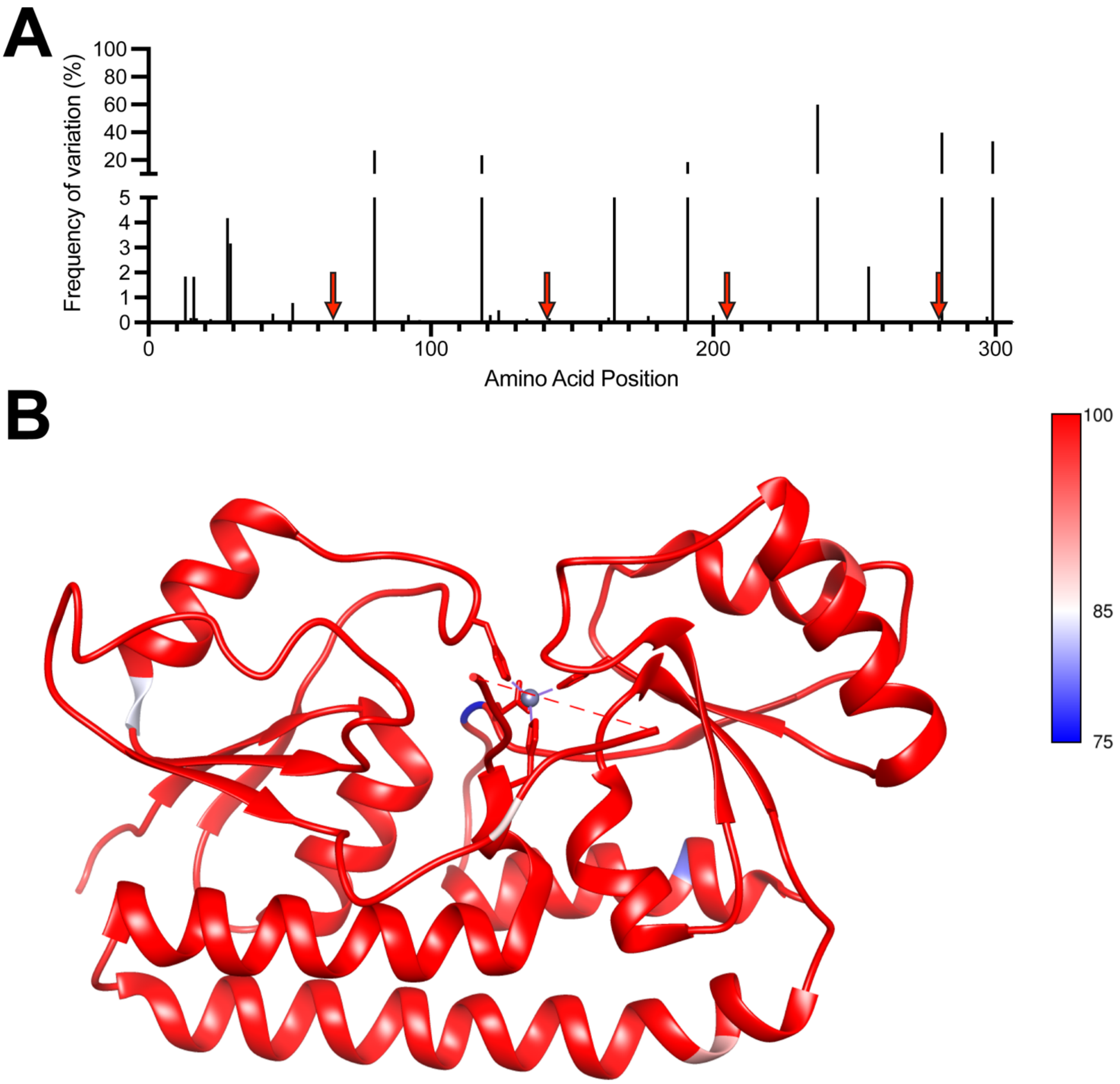

Supplementary Figure 1. Sequence conservation analysis of AdcAII amino acid sequence based on the non-redundant amino acid sequences from 20,016 *S. pneumoniae* genomes. (A) Frequency of variation (% of sequences differing from the reference strain [serotype 2 D39] / 20,016) for each residue of AdcAII from 20,016 non-redundant amino acid sequences. Metal-binding residues are indicated by red arrows. **(B)** Amino acid residue conservation based on non-redundant sequences mapped onto Zn(II)-AdcAII (PDB ID: 3CX3). Residues with 100% conservation are colored red, with 85% in white, and 75% in blue. Zn(II)-coordinating residues are depicted as sticks.

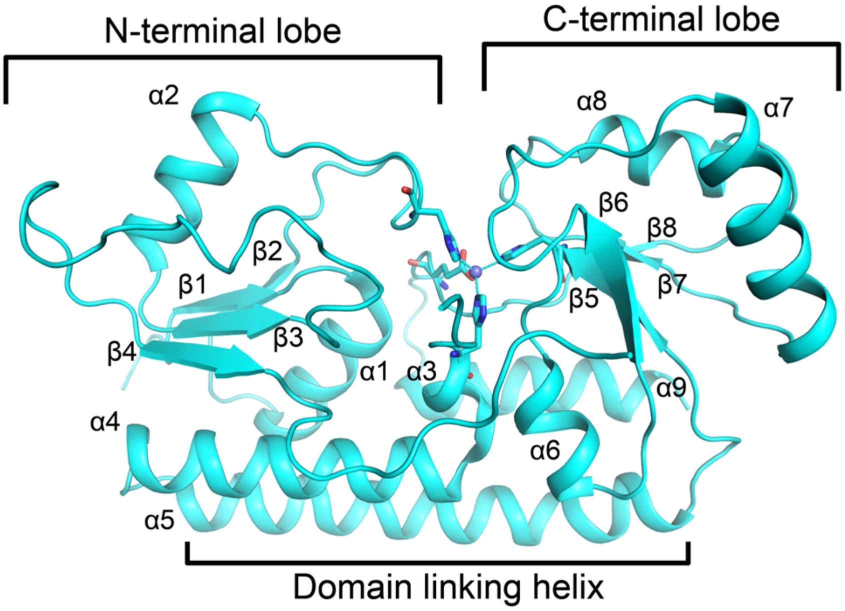

Supplementary Figure 2. AdcAII structure. Cartoon representation of AdcAII viewed from the "side". The bound Zn(II) is shown as a sphere and its coordinating residues as sticks.

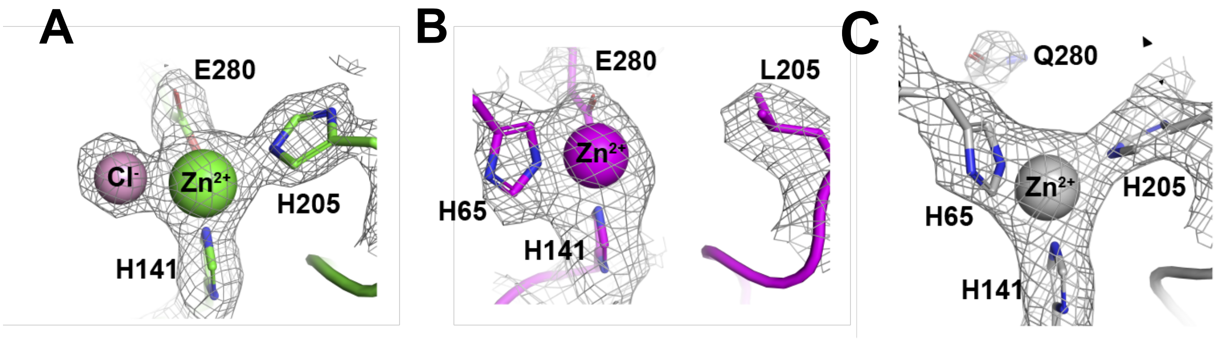

Supplementary Figure 3. Electron density of the metal-binding site in the crystal structure of the AdcAII variant proteins. Zn(II)-coordinating residues are shown as sticks, with ions as spheres for **(A)** $AdcAII_{H65A}$, **(B)** $AdcAII_{H205L}$, and **(C)** $AdcAII_{E280Q}$.

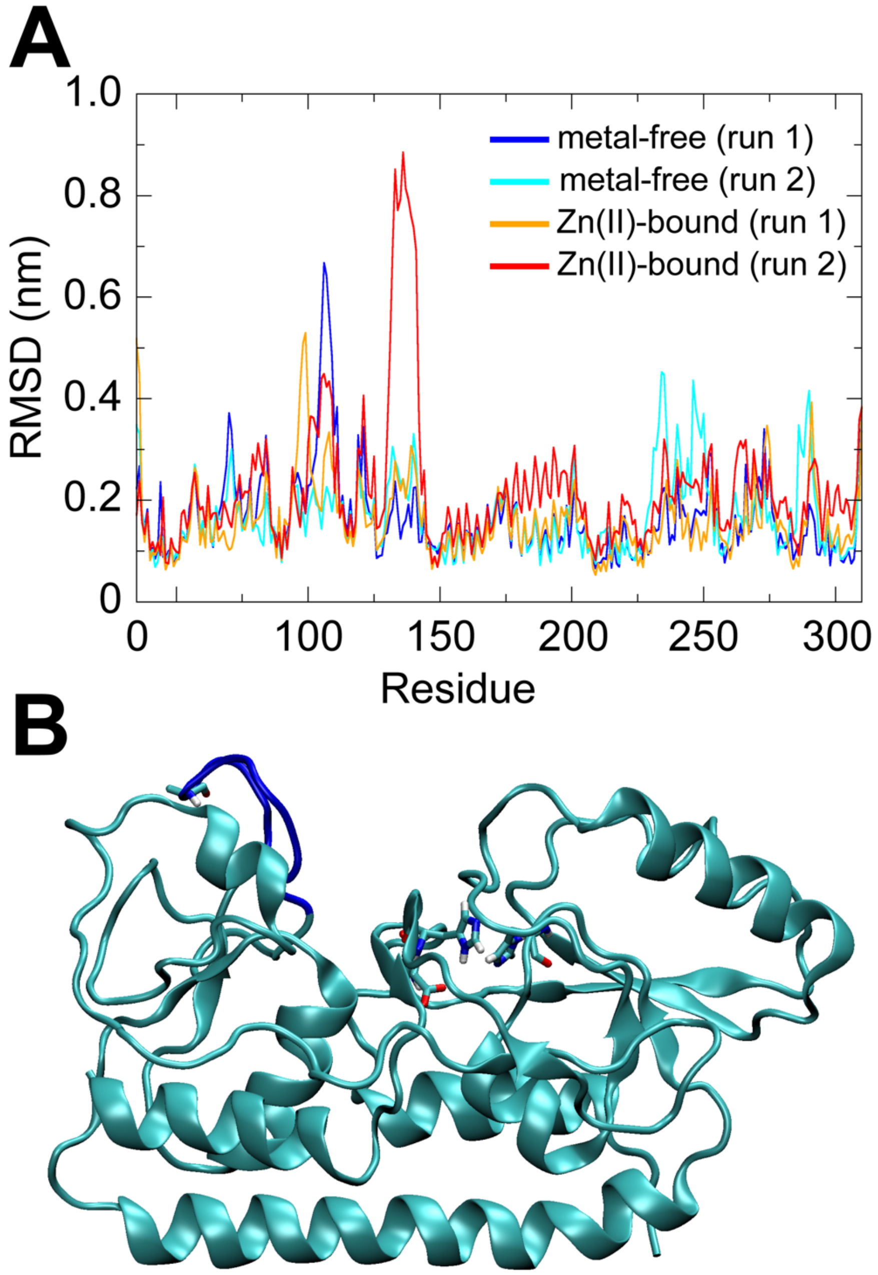

Supplementary Figure 4. Molecular dynamics analyses of AdcAII. (A) Root-mean square fluctuation (RMSF) *vs*. residue for Zn(II)-bound and metal-free AdcAII, obtained from MD simulations**.** RMSF was calculated using the last 250 ns of 750-ns simulations of Zn(II) and metal-free AdcAII. Data for the duplicate runs are shown separately. The RMSF values indicate that the presence of Zn(II) does not affect the mobility of the protein. **(B)** Most populated structure of $AdcAII_{H65A}$ in the Zn(II)-free state as determined from cluster analysis using the last 250 ns of a 750-ns simulation. The protein is shown in cartoon representation in cyan with the α2β2 loop shown in dark blue. Metal-binding residues are show in stick representation.

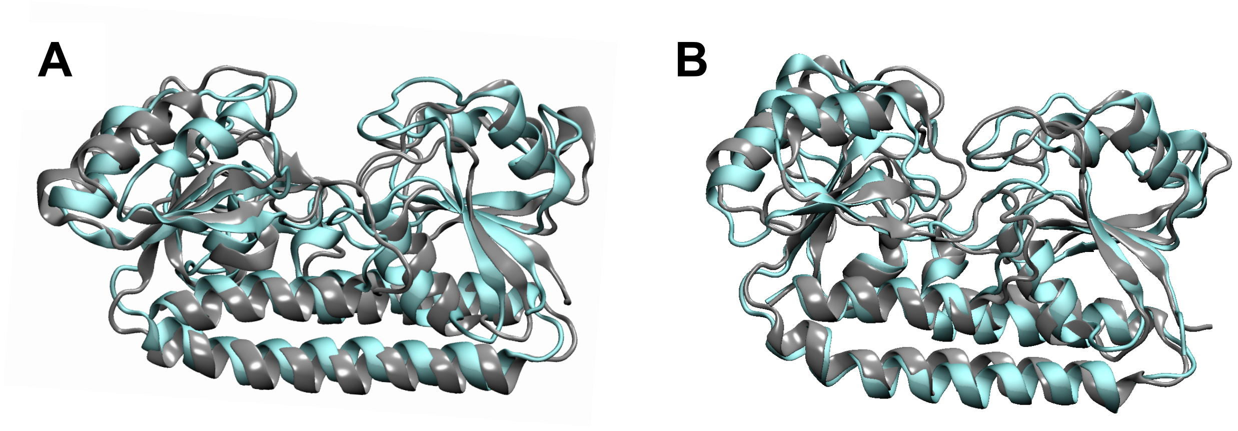

Supplementary Figure 5. Comparison of Zn(II)-bound and metal-free structures of AdcAII$_{H205L}$ and AdcAII$_{E280Q}$. Representative conformations, as determined by clustering analysis, from duplicate 750 ns simulations of metal-free and Zn(II)-bound **(A)** AdcAII$_{H205L}$ and **(B)** AdcAII$_{E280Q}$. The Zn(II)-bound protein is shown in grey and the metal-free bound protein in cyan.

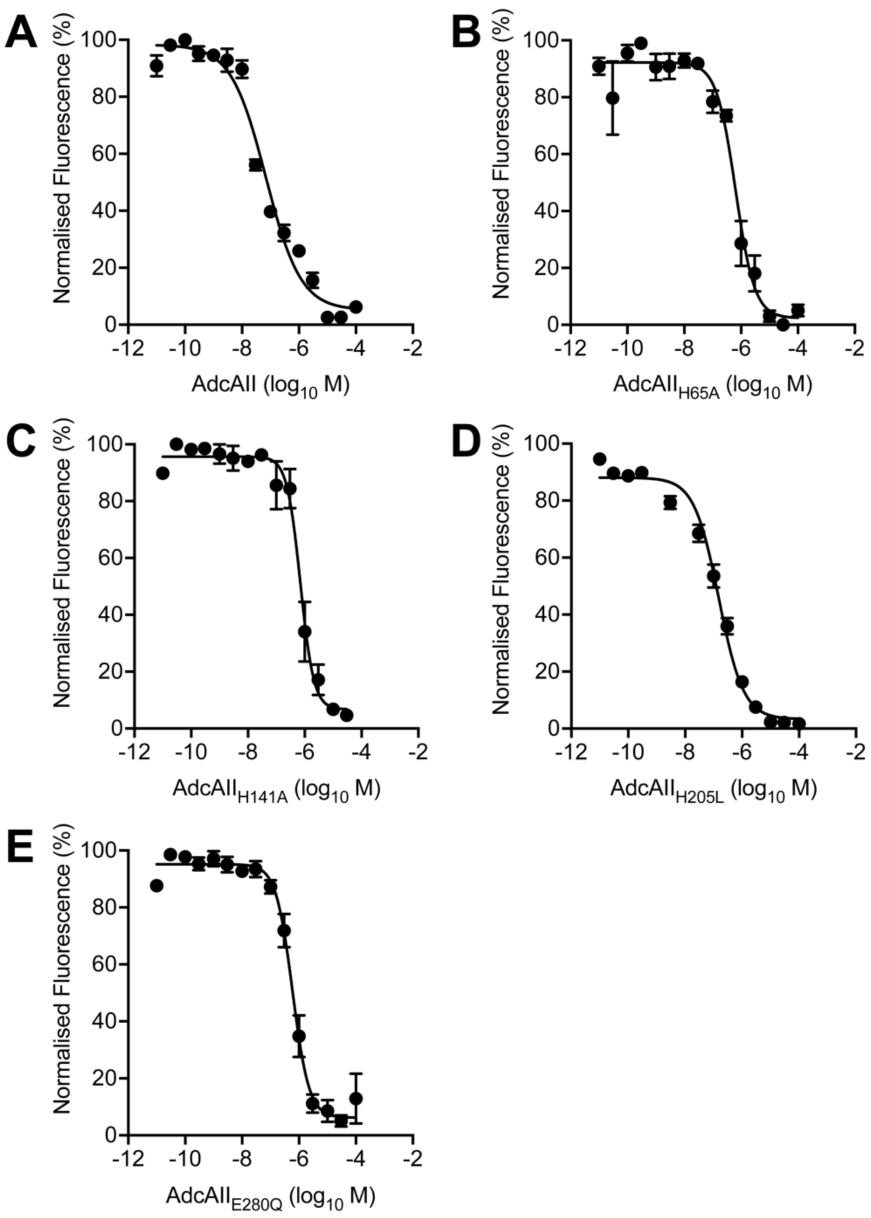

Supplementary Figure 6. Competitive binding assay with AdcAII and mutant variants. Representative analyses of competitive Zn(II) binding by metal-free **(A)** AdcAII, **(B)** AdcAII$_{H65A}$, **(C)** AdcAII$_{H141A}$, **(D)** AdcAII$_{H205L}$, and **(E)** AdcAII$_{E280Q}$ from Mag-Fura-2-Zn(II). Metal-free proteins were titrated against 150 nM Mag-Fura-2-Zn(II) until fluorescence was quenched. The fluorescence data was normalized using experimentally observed fluorescence minimum and maximum values. Each data point corresponds to the representative mean (± S.E.M.) of at least three independent experiments.

SUPPLEMENTARY TABLES

Supplementary Table 1. Prevalence and conservation of multilocus sequence typing (MLST) and cell surface proteins assessed for 20,020 *S. pneumoniae* genomes.

**MLST protein**	**Prevalence (%)**	**Conservation (%)**
spd_1210 (AroE)	99.57	99.50
spd_1158 (GdhA)	99.80	97.26
spd_0580 (Gki)	99.95	99.08
spd_1484 (Ddl)	99.94	99.05
spd_1628 (Xpt)	99.42	99.29
**Surface protein**	**Prevalence (%)**	**Conservation (%)**
spd_2017 (PspC)	43.71	90.73
spd_0126 (PspA)	18.68	84.48
spd_0888 (AdcAII)	99.97	99.25

Supplementary Table 2. X-ray crystallography: data collection, processing and refinement statistics.

	H65A-AdcAII	H205L-AdcAII	E280Q-AdcAII
**Data collection**			
Space group	C 1 2 1	P 6_5 2 2	C 1 2 1
Cell dimensions			
a, *b*, *c* (Å)	46.2, 72.0, 82.0	119.8, 119.8, 169.5	47.3, 72.9, 81.5
α, β, γ (°)	90.0, 95.4, 90.0	90.0, 90.0, 120.0	90.0, 95.6, 90.0
Wavelength (Å)	0.954	0.954	0.954
Resolution (Å)	36.16-2.40 (2.49-2.40)	49.6-3.37 (3.64-3.37)	39.58-3.12 (3.23-3.12)
R_{merge}	0.044 (0.298)	0.192 (0.581)	0.043 (0.19)
$CC_{1/2}$	0.998 (0.871)	0.997 (0.931)	0.996 (0.870)
Average I / σ(I)	16.9 (2.7)	15.0 (6.4)	11.8 (3.9)
Completeness (%)	99.6 (97.0)	99.5 (98.0)	98.0 (88.0)
Multiplicity	7.5	15.6	4.0
**Refinement**			
Resolution (Å)	36.16-2.40 (2.49-2.40)	49.6-3.37 (3.64-3.37)	39.58-3.12(3.23-3.12)
No. unique reflections	10512 (1013)	10715 (2118)	4870 (412)
R_{work} / R_{free} (%)	23.3/26.9	22.9/24.7	22.2/26.0
No. atoms			
Protein	2102	2067	2133
Ligand/ion	14	12	11
Water	60	0	0
B-factors (Å^2)			
Protein	50.2	74.9	57.4
Ligand/ion	49.1	114.5	73.0
Water	43.9	n/a	n/a
R.m.s deviations			
Bond lengths (Å)	0.007	0.008	0.006
Bond angles (°)	0.82	0.98	0.77

Supplementary Table 3. RMSD values from simulations of Zn(II)-bound and metal-free structures of AdcAII$_{E280Q}$.

**Simulation**	**RMSD[a]**
AdcAII$_{E280Q}$ Zn(II)-bound, run1	2.3 ± 0.6
AdcAII$_{E280Q}$ Zn(II)-bound, run2	2.6 ± 0.6
AdcAII$_{E280Q}$ metal-free, run1	2.9 ± 0.2
AdcAII$_{E280Q}$ metal-free, run2	2.9 ± 0.3

a. Average RMSD were calculated from the last 250 ns of the relevant trajectories using back bone atoms only, with the average structure as the reference. Uncertainties are standard deviations. There is no statistically significant difference between the Zn(II)-bound and metal-free structures.

Supplementary Table 4. RMSD values from simulations of Zn(II)-bound and metal-free structures of AdcAII$_{H205L}$.

Simulation	RMSD[a]
AdcAII$_{H205L}$ Zn(II)-bound, run1	3.7 ± 0.6
AdcAII$_{H205L}$ Zn(II)-bound, run2	3.2 ± 0.6
AdcAII$_{H205L}$ metal-free, run1	3.4 ± 0.2
AdcAII$_{H205L}$ metal-free, run2	4.0 ± 0.3

a. Average RMSD were calculated from the last 250 ns of the relevant trajectories using back bone atoms only, with the average structure as the reference. Uncertainties are standard deviations. There is no statistically significant difference between the Zn(II)-bound and metal-free structures.

Supplementary Table 5. ***S. pneumoniae*** **strains used in this study.**

**Strain**	**Genotype**	**Source**
Streptococcus pneumoniae D39	Wild type capsular serotype 2 strain	NCTC7466
D39 *rpsL*$^{+}$	Replacement of *rpsL* gene with K56N mutant variant	Sung et al., 2001
D39 *rpsL*$^{+}$ Δ*adcA::Chl*^{R}	Replacement of *adcA* with chloramphenicol resistance	Plumptre et al., 2014
D39 *rpsL*$^{+}$ Δ*adcA adcAII::Janus*	Replacement of *adcA* with chloramphenicol resistance, replacement of *adcAII* with Janus cassette	This study
D39 *rpsL*$^{+}$ Δ*adcA* Ω*adcAII*$_{H65A}$	Replacement of *adcA* with chloramphenicol resistance, replacement of *adcAII* with *adcAII*$_{H65A}$ variant	This study
D39 *rpsL*$^{+}$ Δ*adcA* Ω*adcAII*$_{H141A}$	Replacement of *adcA* with chloramphenicol resistance, replacement of *adcAII* with *adcAII*$_{H141A}$ variant	This study
D39 *rpsL*$^{+}$ Δ*adcA* Ω*adcAII*$_{H205L}$	Replacement of *adcA* with chloramphenicol resistance, replacement of *adcAII* with *adcAII*$_{H205L}$ variant	This study
D39 *rpsL*$^{+}$ Δ*adcA* Ω*adcAII*$_{E280Q}$	Replacement of *adcA* with chloramphenicol resistance, replacement of *adcAII* with *adcAII*$_{E280Q}$ variant	This study

Supplementary Table 6. Oligonucleotide primers used in this study.

Primer	Sequence (5' → 3')	Purpose
AdcAII_GA1F	GGAAAGGGGATGAAAATTGTGACCAGT	Cloning of *adcAII* gene into pCAM-cLIC01 vector by Gibson assembly
AdcAII_GA1R	CTTTAATTCTTCTGCTAGAATACTCATATTTTCTTCAAGATTTTCTAA	As above
AdcAII_GA2F	GGAAAGGGGATGAAAATTGTGACCAGT	As above
AdcAII_GA2R	CTTTAATTCTTCTGCTAGAATACTCATATTTTCTTCAAGATTTTCTAA	As above
AdcAII_H65A_1F	TTCGGATGATTCAGTCAAGTAGTGGTATTGCGTCCTTTGAACCTTCG	Site-directed mutagenesis of AdcAII His65 to Ala
AdcAII_H65A_1F	CGAAGGTTCAAAGGACGCAATACCACTACTTGACTGAATCATCCGAA	As above
AdcAII_H141A 1F	TGATGAAAAAACGCTCTATGACCCTGCGACATGGCTAGATCCTGAA	Site-directed mutagenesis of AdcAII His141 to Ala
AdcAII_H141A 1R	TTCAGGATCTAGCCATGTCGCAGGGTCATAGAGCGTTTTTTCATCA	As above
AdcAII_H205L_1F	CGCTAGATAAGAAAAGGCTGTAAGTTGTGTTACAAATGTTTTCTG	Site-directed mutagenesis of AdcAII His205 to Leu
AdcAII_H205L_1R	CAGAAAACATTTGTAACACAACTTACAGCCTTTTCTTATCTAGCG	As above
AdcAII_E280Q_1F	TGTGGGTCTGACTGTAAAGGATTCAGAGTTTTAAGACCC	Site-directed mutagenesis of AdcAII Glu280 to Gln
AdcAII_E280Q_1R	GGGTCTTAAAACTCTGAATCCTTTACAGTCAGACCCACA	As above
Janus_F	CCGTTTGATTTTTAATGGATAATG	Amplification of Janus cassette
Janus_R	AGAGACCTGGGCCCCTTTCC	As above
adcAII_seq_F	TGTGAACACCTGGATTGCTT	Sequencing and colony PCR of mutant *adcAII* inserts
adcAII_seq_R	TGGTCTCCATGAGAGGTCAC	As above
adcAII_Flank_F	TACCAAAGCAACCACCTTCC	Overlap extension PCR to join upstream flank of *adcAII* with *adcAII* insert and downstream flank
adcAII_Flank_R	TGGTTTTTCCGTTTGATTGG	As above
adcAII_Janus_X	CATTATCCATTAAAAATCAAACGGGAACAACTCCTTAACCATTTAATTAA	Amplification of *adcAII* flanks with overlapping regions complementary to Janus cassette

adcAII_Janus_Y	AGGGGCCCAGGTCTCTGGAAAGAATGAAAATCAATAAAAAAT	As above
adcAII_F	GGAAAGGGGATGAAAATTGTGAC	Amplification of mutant *adcAII* inserts from expression constructs (Supplementary Table 5)
adcAII_R	CTTTAATTCTTCTGCTAGAATACTCATATT	As above
adcAII_X	TTTTCATCCCCTTTCCTGTCTG	Amplification of upstream flank of *adcAII*, in combination with primer adcAII_F
adcAII_Y	CTAGCAGAAGAATTAAAGTGAGG	Amplification of downstream flank of *adcAII*, in combination with primer adcAII_R

Supplementary Table 7. Expression constructs used in this study

Plasmid	Description	Source
pCAM-cLIC01	KanR, ligation independent cloning expression vector encoding a C-terminal dodecahistidine tag	Plumptre et al., 2014
pCAM-cLIC01-AdcAII	KanR, *adcAII* gene lacking the signal sequence (residues 1-28), cloned into pCAM-cLIC01 vector	This study
pCAM-cLIC01-AdcAII-H65A	KanR, *adcAII* gene lacking the signal sequence with a H65A mutation, cloned into pCAM-cLIC01 vector	This study
pCAM-cLIC01-AdcAII-H141A	KanR, *adcAII* gene lacking the signal sequence with a H141A mutation, cloned into pCAM-cLIC01 vector	This study
pCAM-cLIC01-AdcAII-H205L	KanR, *adcAII* gene lacking the signal sequence with a H205L mutation, cloned into pCAM-cLIC01 vector	This study
pCAM-cLIC01-AdcAII-E280Q	KanR, *adcAII* gene lacking the signal sequence with a E280Q mutation, cloned into pCAM-cLIC01 vector	This study
